# Integration of genetic, transcriptomic, and clinical data provides insight into 16p11.2 and 22q11.2 CNV genes

**DOI:** 10.1186/s13073-021-00972-1

**Published:** 2021-10-29

**Authors:** Mikhail Vysotskiy, Xue Zhong, Tyne W. Miller-Fleming, Dan Zhou, Autism Working Group of the Psychiatric Genomics Consortium^, Bipolar Disorder Working Group of the Psychiatric Genomics Consortium^, Schizophrenia Working Group of the Psychiatric Genomics Consortium^, Nancy J. Cox, Lauren A. Weiss

**Affiliations:** 1 Department of Psychiatry and Behavioral Sciences, University of California San Francisco, 513 Parnassus Ave., Health Sciences East 9th floor HSE901E, San Francisco, CA 94143 USA; 2 Institute for Human Genetics, University of California San Francisco, San Francisco, CA 94143 USA; 3 Weill Institute for Neurosciences, University of California San Francisco, San Francisco, CA 94143 USA; 4 Pharmaceutical Sciences and Pharmacogenomics Graduate Program, University of California San Francisco, San Francisco, CA 94143 USA; 5 Division of Genetic Medicine, Department of Medicine, Vanderbilt University Medical Center, Nashville, TN 37232 USA; 6 Vanderbilt Genetics Institute, Nashville, TN 37232 USA

**Keywords:** Copy number variants, Transcriptome imputation, Electronic health records, Psychiatric traits, Phenome-wide association studies

## Abstract

**Background:**

Deletions and duplications of the multigenic 16p11.2 and 22q11.2 copy number variant (CNV) regions are associated with brain-related disorders including schizophrenia, intellectual disability, obesity, bipolar disorder, and autism spectrum disorder (ASD). The contribution of individual CNV genes to each of these identified phenotypes is unknown, as well as the contribution of these CNV genes to other potentially subtler health implications for carriers. Hypothesizing that DNA copy number exerts most effects via impacts on RNA expression, we attempted a novel in silico fine-mapping approach in non-CNV carriers using both GWAS and biobank data.

**Methods:**

We first asked whether gene expression level in any individual gene in the CNV region alters risk for a known CNV-associated behavioral phenotype(s). Using transcriptomic imputation, we performed association testing for CNV genes within large genotyped cohorts for schizophrenia, IQ, BMI, bipolar disorder, and ASD. Second, we used a biobank containing electronic health data to compare the medical phenome of CNV carriers to controls within 700,000 individuals in order to investigate the full spectrum of health effects of the CNVs. Third, we used genotypes for over 48,000 individuals within the biobank to perform phenome-wide association studies between imputed expressions of individual 16p11.2 and 22q11.2 genes and over 1500 health traits.

**Results:**

Using large genotyped cohorts, we found individual genes within 16p11.2 associated with schizophrenia (*TMEM219*, *INO80E*, *YPEL3*), BMI (*TMEM219*, *SPN*, *TAOK2*, *INO80E*), and IQ (*SPN*), using conditional analysis to identify upregulation of *INO80E* as the driver of schizophrenia, and downregulation of *SPN* and *INO80E* as increasing BMI. We identified both novel and previously observed over-represented traits within the electronic health records of 16p11.2 and 22q11.2 CNV carriers. In the phenome-wide association study, we found seventeen significant gene-trait pairs, including psychosis (*NPIPB11*, *SLX1B*) and mood disorders (*SCARF2*), and overall enrichment of mental traits.

**Conclusions:**

Our results demonstrate how integration of genetic and clinical data aids in understanding CNV gene function and implicates pleiotropy and multigenicity in CNV biology.

**Supplementary Information:**

The online version contains supplementary material available at 10.1186/s13073-021-00972-1.

## Background

Multi-gene copy number variants (CNVs), including a 600-kb region at 16p11.2 and a 3-Mb region at 22q11.2, are known causes of multiple brain-related disorders. The 16p11.2 CNV, originally identified as a risk factor for autism spectrum disorder (ASD), has also been associated with schizophrenia, bipolar disorder, intellectual disability, and obesity [1–5]. The 22q11.2 CNV, identified as the cause of DiGeorge (velocardiofacial) syndrome, is associated with schizophrenia, intellectual disability, obesity, bipolar disorder, and ASD, as well [6–11]. The effects of these two CNVs can be further subdivided into the effects of deletions vs. duplications. Some disorders are shared among carriers of deletions and duplications of the same region, and others show opposite associations. For instance, ASD and intellectual disability are observed in both deletion and duplication carriers in both 16p11.2 and 22q11.2 [3–8, 12–14]. Other traits are specific to one direction of the copy number change: schizophrenia and bipolar disorder are observed in 16p11.2 duplication carriers, but not deletion carriers [2]. A third category of 16p11.2- and 22q11.2-associated traits are “mirrored”. 16p11.2 deletion carriers show increased rates of obesity, while duplication carriers tend to be underweight. 22q11.2 duplication carriers show reduced rates of schizophrenia, as opposed to increased rates in deletion carriers [1, 15, 16]. The question of which specific genes drive which brain-related traits associated with 16p11.2 or 22q11.2 CNVs remains unanswered. Likewise, what else these genes might be doing has been difficult to assess in small numbers of identified CNV carriers, who are primarily children. Identifying the role of specific gene(s) in behavioral and medical traits will clarify the biological processes that go awry as a result of these CNV mutations and the mechanisms by which they do so. Knowledge of the genes and mechanisms involved would, in turn, provide opportunities to develop targeted treatments.

Three of the traditional ways to map CNV genes to disorders are identifying loss-of-function mutations in these genes, analyzing smaller subsets of the entire region, and finding mutations in animal models that are sufficient to recapitulate the phenotype. The loss-of-function mutation method was used to fine-map the 17p11.2 CNV, another CNV associated with behavioral and non-behavioral traits [17, 18]. Most of the features of the deletion syndrome, including intellectual disability, are represented in individuals who carry a defective copy of the *RAI1* gene due to point mutation [19]. Duplications of *Rai1* appear to explain body weight and behavior abnormalities in mouse models of 17p11.2 duplications [20]. Another example is the Williams syndrome CNV at 7q11.23 [21, 22]. The cardiac traits associated with this syndrome are present in individuals with only one functional copy of the *ELN* gene, but this gene does not explain the behavioral traits [23, 24]. The second method, of finding a smaller “critical region,” was used to fine-map the 17q21.31 CNV [25, 26]. By comparing patients who had similar symptoms with overlapping cytogenetic profiles, the common breakpoints of the CNV region were refined to a region containing only six genes [26]. Later, Koolen et al. identified patients showing intellectual disability and facial dysmorphisms characteristic of this CNV with disruptive mutations in one of the six genes, *KANSL1* [27]. The third method of recapitulating similar phenotypes in animal models was successful in identifying *TBX1* as a gene important for some of the physical traits involved with 22q11.2 deletions. Mice with heterozygous mutations in the *TBX1* gene show cardiac outflow tract anomalies, similar to human 22q11.2 deletion carriers [28–30]. However, it is unclear that *TBX1* is sufficient to explain brain-related disorders in 22q11.2 carriers [31, 32].

The 16p11.2 and 22q11.2 CNVs have been resistant to these traditional approaches for fine-mapping brain-related traits. To date, no highly penetrant point mutations in 16p11.2 or 22q11.2 genes have been shown to be sufficient for a brain-related disorder. The most recent schizophrenia GWAS from the Psychiatric Genomics Consortium discovered a common SNP association near the 16p11.2 region; however, the specific genes underlying GWAS signals are often unknown [33]. No small subsets of 16p11.2 or 22q11.2 genes have been proven necessary and sufficient to cause a brain-related disorder. A subregion of 22q11.2 has been proposed to explain ASD associated with deletions [34]. As this subset of 22q11.2 contains approximately 20 genes, it is likely that further fine-mapping within this subset is possible. At 16p11.2, a subset of five deleted genes was isolated in a family with a history of ASD [35]. However, this mutation neither caused ASD in all deletion carriers, nor was responsible for ASD in some non-carrier family members. Non-human models for the 16p11.2 and 22q11.2 CNVs, as well as knockouts for individual genes are available in mouse, zebrafish, and fruit flies [36–41], but have not successfully mapped individual genes in these CNVs to brain-related traits [28–30]. Different zebrafish studies of 16p11.2 homologs have implicated different genes as phenotype drivers, as well as shown that most were involved in nervous system development [37, 38, 42]. The complex brain-related traits associated with these CNVs are unlikely to be fully captured in model organisms. Hallucinations, a common symptom of schizophrenia, can be identified only in humans. There may be other aspects of 16p11.2 and 22q11.2 CNV biology that are human-specific. For example, mice carrying 16p11.2 duplications are obese, while obesity is associated with deletions in humans [43]. Given the insufficiency of previous approaches, new approaches for fine-mapping genes in these regions for brain-related traits are necessary.

The motivation behind our approach is that in 16p11.2 and 22q11.2 CNV carriers, variation in gene copy number is expected to lead to variation in RNA expression level (with downstream effects on protein product). Expression measurements in mouse or human cell lines carrying 16p11.2 and 22q11.2 deletions and duplications confirm that for nearly all genes, duplication carriers have increased expression of individual CNV genes compared to controls, and deletion carriers have reduced expression compared to controls [44–49]. As the breakpoints of these CNVs are unlikely to cause gain-of-function, we believe that the variation in expression of one or more of the genes in/near the CNV is the cause of pathogenicity. While these CNVs significantly disrupt gene expression levels, most genes’ expression levels vary among the general population, sometimes by a factor of two or more, as studies such as the Genotype-Tissue Expression Consortium (GTEx) have shown [50–53]. This variation can be, in part, attributed to common genetic polymorphisms (expression quantitative trait loci, eQTLs). If large expression deviation in duplication and deletion carriers is a risk factor for a disorder, we hypothesize that more modest expression variation in the same genes among non-carriers will be a modest risk factor for the same disorder or milder related traits. This idea is analogous to the well-supported observation that common polymorphisms of small effect associated with a common trait can overlap with Mendelian genes for a similar trait [54–56].

**Fig. 1:**
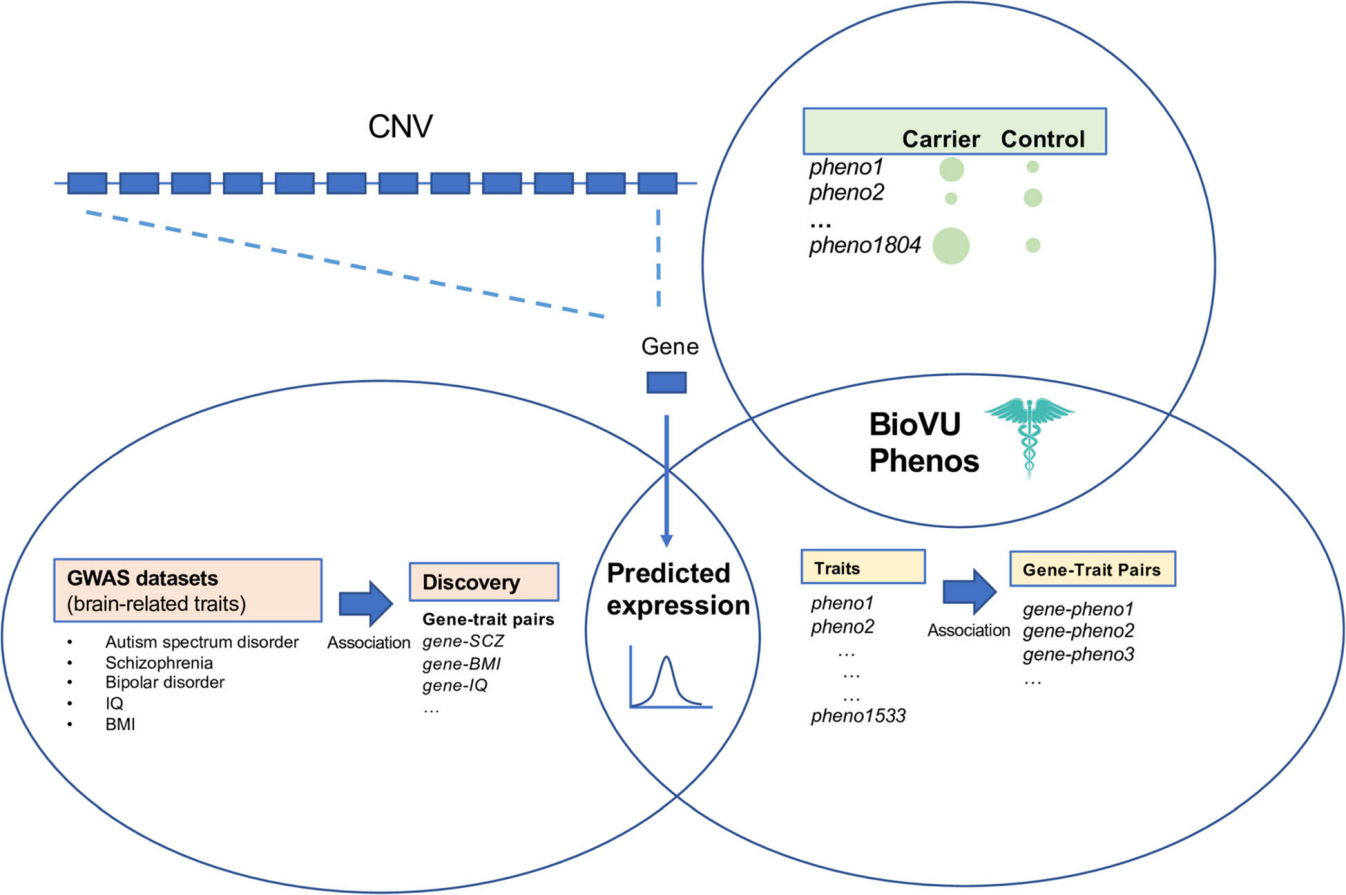
An overview of the three components of this study. We probed the effects of individual genes in the 16p11.2 and 22q11.2 CNVs on phenotype in two ways. First (bottom left), we used large GWAS datasets for brain-related traits associated with both CNVs to determine whether variation in predicted expression in any of the individual genes in each CNV was associated with case-control status for each trait. In the second component of this study (top right), we used a biobank containing clinical and genotypic data to identify the individuals with 16p11.2 and 22q11.2 duplications or deletions and determined the clinical traits that were over-represented in CNV carriers. Third (bottom right), we used the biobank to perform a phenome-wide association study to determine clinical traits that are driven by the predicted expression of individual CNV genes, as well as whether these traits overlapped with traits over-represented in CNV carriers. Analyses 1 and 3 are integrated in their use of imputed expression; analyses 2 and 3 are integrated in their use of electronic health data

Here, we perform three in silico studies of the impact of predicted expression of individual 16p11.2 and 22q11.2 genes, in comparison with the diagnosed CNVs, on human traits (Fig. 1). First, we identify genes associated with brain-related disorders via expression variation. Recent tools have leveraged the heritability of gene expression, allowing us to “impute” gene expression for genotyped individuals using eQTLs [57, 58]. We perform association testing between imputed expression and five brain-related traits common to the 16p11.2 and 22q11.2 CNVs for which large amounts of genetic data have been amassed: schizophrenia, IQ, BMI, bipolar disorder, and ASD [59–63]. We find at least one 16p11.2 gene associated with schizophrenia, IQ, and BMI. Second, we use BioVU, a biobank containing electronic health records (EHRs) for over 3 million individuals, to determine the medical traits in CNV carriers detected in our EHR system, confirming canonical CNV features and discovering novel over-represented traits [64]. We also probe the consequences of expression variation of individual 16p11.2 and 22q11.2 genes on the medical phenome, by imputing gene expression in the > 48,000 genotyped individuals in the BioVU health system and performing a phenome-wide association test across all available traits. We find that mental disorders are over-represented among top gene-trait association pairs, and we highlight genes associated with the traits over-represented in CNV carriers. Taken together, our work provides a comprehensive catalog of associations of individual CNV genes to traits across the phenome.

## Methods

### GWAS data for schizophrenia, IQ, BMI, bipolar disorder, and ASD

We obtained the imputed individual-level genotypes for ASD, bipolar disorder, and schizophrenia from the Psychiatric Genomics Consortium in PLINK format (Additional file 1: Table S1). These datasets include mainly European populations and are comprised of several independent cohorts: 30 in bipolar disorder (*N* = 19,202 cases 30,472 controls, downloaded July 2019), 46 in schizophrenia (*N* = 31,507 cases 40,230 controls, downloaded July 2018), 14 in ASD (*N* = 7386 cases, 8566 controls, downloaded May 2019) [60, 61, 65]. For two additional traits, we used publicly available summary statistics: BMI from the Genetic Investigation of ANthropometric Traits (GIANT) consortium (2015, both sexes, *n* = 339,224, downloaded June 2019) and IQ from Savage et al. (2018) hosted by the Complex Trait Genomics lab at VU Amsterdam (*n* = 269,867, downloaded May 2019) [62, 63].

For replication studies and comparison of PheWAS results, we used the publicly available GWAS summary statistics for schizophrenia, IQ, BMI, bipolar disorder, and ASD from the UK Biobank [66]. We could not use the UK Biobank IQ data for replication of our discovery IQ data, as the datasets overlap. The list of UK Biobank phenotypes used is in Table S1 in Additional file 1. In addition, we used individual-level data from the UK Biobank (*n* = 408,375) to perform conditional analysis for BMI fine-mapping, but chose not to use it for discovery analysis because of previously observed high inflation of summary statistics [67, 68].

### Expression prediction models

In order to impute gene expression, we obtained PrediXcan models for 48 tissues based on GTEx v7 Europeans [57, 58, 69]. These models were generated by training an elastic net model that finds the best set of cis-SNP predictors for the expression of a gene in a tissue in the GTEx genotyped individuals [57]. Only models with predicted-observed correlation *R*^2^ > 0.01 and cross-validated prediction performance *P* < 0.05 are kept.

### Genes studied

We studied all coding and noncoding genes at the 16p11.2 and 22q11.2 copy number variant loci for which expression prediction models were available. We included flanking genes in a 200-kb window upstream and downstream of the CNV breakpoints. Overall, 37 coding and 8 noncoding genes at or near 16p11.2, as well as 52 coding and 30 noncoding genes at or near 22q11.2, were tested. Not all genes in the CNV regions were available to be analyzed through our methods; noncoding genes were especially unlikely to have a high-quality predictive model in any tissue. Thirty-four genes (of which 27 were noncoding) at or near 16p11.2 lacked high-quality prediction models in every tissue. One hundred two genes (of which 90 were noncoding) at or near 22q11.2 lacked high-quality prediction models in every tissue. (Additional file 2:Table S2, Additional file 3: Fig. S1).

### Comparison of observed expression correlations with predicted expression correlations

Observed expression correlations were calculated at a tissue-specific level on data from GTEx v7 [70]. Tissue-specific predicted expression was calculated by applying the appropriate GTEx predictive model on the GTEx v6p genotypes (dbgap id: phs000424.v6.p1) for 450 individuals. To minimize spurious correlations, the predicted expression levels were rigorously filtered and normalized. Specifically, the expression levels were filtered for outliers (values above 1.5 × interquartile range, in either direction), adjusted for the principal components of both the predicted expression levels and the first 20 PCs of the GTEx genotypes, inverse-quantile normalized, re-adjusted for principal components, and re-filtered for outliers. We observed that normalization of the predicted expression reintroduced correlation between expression and the genotypic PCs, leading us to perform the correction twice.

### Association analysis in individual-level data

Each of the three PGC collections went through quality control, filtering, and PCA calculation, as described previously [59–61]. In each individual cohort, the convert_plink_to_dosage.py script in PrediXcan was used to convert chromosome 16 and 22 genotypes from PLINK to dosage format, keeping only the SNPs used in at least one predictive model. Using these dosages, the --predict function in PrediXcan was used to generate predicted expressions of CNV genes for each individual. Genes with predicted expression of 0 for all individuals in a single tissue were filtered out. The average number of genes filtered out across tissue-cohort pairs was 0.89; the maximum was 11 in thyroid tissue in the Japanese schizophrenia cohort. Cross-tissue association studies between predicted expression and case-control status were performed using MultiXcan. In brief, MultiXcan takes the matrix of predicted expressions across tissues for a single gene, calculates the principal components of this matrix to adjust for collinearity, fits a model between these PCs and case-control status, and reports the results of the overall fit [58]. As in the PGC association studies, our analysis was adjusted by the principal components that were significantly associated with each trait—7 for bipolar disorder, 10 for schizophrenia, and 8 for autism case-control studies (the autism trios were not adjusted for covariates). UK Biobank MultiXcan analysis was limited to individuals who reported their ethnicity as “white,” and included age, age-squared, and 40 principal components as covariates.

Meta-analysis with METAL on the *p* values from MultiXcan, weighted by the sample size of each cohort, was used to calculate a cross-cohort association statistic for each trait individually [71]. The joint fit in MultiXcan generates an F-statistic that is always greater than zero, while some of our traits of interest have a specific expected direction (only seen in deletion carriers or only seen in duplication carriers). Thus, a direction was assigned to each MultiXcan result. This was done by running a tissue-specific PrediXcan association analysis between predicted expressions and case-control status (using --logistic), which calculates a signed association *Z*-score for every gene. The sign of the mean *Z*-score for that gene across all tissues was the direction of association used for meta-analysis.

### Association analysis in summary-level data

Both the single-tissue PrediXcan and the multitissue MultiXcan methods have been extended to estimate the association results between genetically regulated expression and a trait if only the summary statistics for the trait are available. For each trait’s summary statistics, the summary version of PrediXcan (S-PrediXcan) and the associated MetaMany.py script was used to calculate the per-tissue association results for each gene in 48 GTEx tissues. Association results were aggregated across tissues using the summary version of MultiXcan (S-MultiXcan). The mean single-tissue *Z*-score (as reported in the zmean column in the S-MultiXcan output) was used as the direction of association. The UK Biobank replication studies were performed in the same way.

### Conditional analysis to fine-map associations

Existing methods for fine-mapping PrediXcan associations (such as FOCUS [72] and MR-JTI [73]) are tissue-specific and focus on summary statistics. Given that we have individual-level data and use a cross-tissue approach, we chose to use a conditional analysis approach. In order to adapt the multitissue association analysis to perform conditional testing, “conditioned predicted expressions” were generated for a set of genes associated with the same trait. As an example, take the set of three genes [*INO80E*, *YPEL3*, *TMEM219*] associated with schizophrenia. In order to condition on *INO80E*, for example, the predicted expression of *INO80E* was regressed out of the predicted expressions of *YPEL3* and *TMEM219*. Conditioning was only done in tissues where the predicted expressions of the genes were correlated (Spearman correlation *P* < 0.05). Another set of conditioned predicted expressions was generated by adjusting the predicted expression of *INO80E* by the predicted expressions of [*TMEM219*, *YPEL3*]. Separately, these per-tissue conditioned predicted expressions were used as inputs for a MultiXcan analysis and METAL meta-analysis on schizophrenia as described earlier. All three individually associated genes were tested in this manner. The same analysis was later used to test for independence of association between BMI in the UK Biobank as well as *psychosis* and *morbid obesity* traits in the PheWAS. The *P*_*cond*_ reported in the text is the *p* value of a gene-trait pair when adjusting for all other genes considered for conditioning for this trait, unless otherwise stated. To validate that our approach explained all GWAS signal at the loci, we also conditioned the MultiXcan analysis on lead GWAS SNP(s) that were also eQTLs. The GWAS conditioning was performed in PLINK using the --condition function, with principal components (and age for BMI) as covariates. Linkage disequilibrium patterns in the region were visualized using LocusZoom [74].

### Phenome-wide association studies

Vanderbilt University Medical Center (VUMC) houses de-identified phenotypic data in the form of the electronic health records (EHR) within the synthetic derivative (SD) system [64]. The SD contains EHR data including ICD9/10 billing codes, physician notes, lab results, and similar documentation for 3.1 million individuals. BioVU is a biobank at VUMC that is composed of a subset of individuals from the SD that have de-identified DNA samples linked to their EHR phenotype information. The clinical information is updated every 1–3 months for the de-identified EHRs. Detailed description of program operations, ethical considerations, and continuing oversight and patient engagement have been published [64]. At time of analysis, the biobank contained 48,725 individuals who had been genotyped. DNA samples were genotyped with genome-wide arrays including the Multi-Ethnic Global (MEGA) array, and the genotype data were imputed into the HRC reference panel [75] using the Michigan imputation server [76]. Imputed data and the 1000 Genome Project data were combined to carry out principal component analysis (PCA) and European ancestry samples were extracted for analysis based on the PCA plot. GTEx v7 models from PredictDB were applied to the samples to calculate genetically regulated expression (GReX).

Phenome-wide association study (PheWAS) was carried out using “phecodes,” phenotypes derived from the International Code for Diseases version 9 (ICD-9) billing codes of EHRs. The PheWAS package for R, version 0.11.2-3 (2017), was used to define case, control, and exclusion criteria [77, 78]. We required two codes on different visit days to define a case for all conditions, and only phecodes with at least 20 cases were used for analysis (1531 traits). The single-tissue predicted expressions were combined across tissues using MultiXcan, as was done to analyze individual-level GWAS data from the Psychiatric Genomics Consortium [58]. Covariates for this analysis were age, sex, genotyping array type/batch and three principal components of ancestry.

The top 1% (top 15 traits) of every gene’s association results were kept for analysis. A binomial test was used to compare whether the number of traits in any clinical category (circulatory system, genitourinary, endocrine/metabolic, digestive, neoplasms, musculoskeletal, injuries and poisonings, mental disorders, sense organs, neurological, respiratory, infectious diseases, hematopoietic, symptoms, dermatologic, congenital anomalies, pregnancy complications) were over-represented in the top 1% of results compared to the proportion of each category among all 1531 traits tested. The expected number of each clinical category as determined by [15 traits × *n*_genes_] × *p*_*i*_ where *p*_*i*_ is the probability of a randomly drawn (without replacement) code belongs to category *i*. *p*_*i*_ can be estimated by the number of codes belonging to category *i* divided by all codes tested (*n* = 1531). The significance threshold was 0.05/[17 categories] = 0.0029.

To analyze the overlap between PheWAS results and known Mendelian phenotypes associated with these genes, we used OMIM [79]. “16p11.2” and “22q11.2” were used as search terms and all CNV gene-trait pairs in the region with OMIM entries were used as the list of expected monogenic traits. For each gene-trait pair in OMIM, relevant similar traits (where available) were identified using the phecode catalog [80] and the top *p* values for these gene-trait pairs in our PheWAS were selected and shown in Additional file 4: Table S3.

### Determining traits over-represented in carriers

3.1 million electronic medical records from the SD at VUMC were queried for keywords corresponding to copy number variants at 16p11.2 and 22q11.2 (Additional file 5: Table S4). Individual charts identified as containing the keywords were manually reviewed and patients were labeled as cases if their medical records provided evidence of CNV carrier status. Patients identified in the queries with insufficient evidence of CNV carrier status were excluded from the analysis. Cases with positive 16p11.2 and 22q11.2 CNV carrier status were identified as: “16p11.2 duplication” (*n* = 48, median age 11), “16p11.2 deletion” (*n* = 48, median age 12), “22q11.2 duplication” (*n* = 43, median age 11). Additional individuals in the 22q11.2 deletion case group were identified by querying the medical records for alternate terms including: “velocardiofacial”, “DiGeorge”, “conotruncal anomaly face,” “Cayler,” “Opitz G/BBB,” “Shprintzen,” and “CATCH22” (*n* = 388, average age 17). Individuals were excluded from case groups if they were included in the genotyped sample used for the gene-by-gene analysis, or if their records included a mention of additional CNVs. Individuals within the 16p11.2 case groups were also excluded if the size of the reported CNV was 200–250 kb. Individuals within the 22q11.2 case group were excluded if the size of the CNV was smaller than 500 kb or if there was a mention of “distal” when referring to the deletion or duplication. PheWAS was carried out, with each of the four carrier categories as cases and over 700,000 medical home individuals as controls, using age, sex, and self-reported race as covariates. The medical home individuals are patients seen at a Vanderbilt affiliated clinic on five different occasions over the course of 3 years. Because the sample size for this analysis was larger (700,000 individuals vs. 48,000), and we used traits that were present in 20 or more individuals, there were more traits available for analysis here, *n* = 1795. After calculating PheWAS, we excluded over-represented traits that were present in < 5% of carriers from further analyses.

### Comparing gene-specific PheWAS to carrier vs. non-carrier PheWAS

For the first comparison, for each of 16p11.2 duplications, 16p11.2 deletions, 22q11.2 duplications, 22q11.2 deletions, the entire carrier vs. non-carrier PheWAS results were ranked. All the traits in the top 1% of per-gene 16p11.2 and 22q11.2 PheWAS results were converted to a value corresponding to the rank of the trait in the carrier vs. non-carrier PheWAS. To determine whether the per-gene PheWAS top traits were distributed nonrandomly with respect to carrier association, the distribution of the ranks of the each CNV’s per-gene PheWAS top traits was compared to the ranks of all carrier vs. non-carrier PheWAS traits for the same CNV (a uniform distribution) using a one-tailed Wilcoxon rank sum test.

For the second comparison, individuals carrying “extreme” predicted expression across a CNV region were identified using a sequence of rankings. Each expression measurement (i.e., the expression of a single gene in a single tissue in a single individual) was classified as “extreme” if it ranked above the top 2nd percentile or below the bottom 2nd percentile of the BioVU cohort, “normal” if the measurement was between the 25th and 75th percentile, or “neither.” For a gene expressed in only one tissue, the gene’s “extreme” expression label is simply the same as the tissue’s “extreme” label. For a gene with multiple tissue expressions, we counted the number of tissues with “extreme” expression and assigned a gene-level “extreme” label to individuals with the most tissues consistently expressing “extremes” for the gene. A gene-level “normal” label was assigned to half of the cohort who had no extreme expression in any tissues and had the most tissues with “normal” expressions. The remaining individuals received a “neither” label for the gene. After obtaining the gene-level labels (“extreme,” “normal,” “neither”), we then ranked the individuals by the number of “extreme” expression genes, and labeled a subset of individuals (top 2% of the 48,600 individuals) as extreme expression carriers. Note that we consider extreme high and extreme low predictions together due to prior data showing that eQTL direction can be specific to cell types or tissues, which our cross-tissue approach cannot distinguish [77]. These were compared to a “control” group defined for each CNV region that included individuals with the fewest extreme-expressed genes and most “normal” expression genes who comprised about half of the cohort. PheWAS was performed to identify over-represented traits between the extreme expression and control groups, analogously to the carrier vs. non-carrier PheWAS. The top 10% most associated traits in each category (16p11.2 extreme, 22q11.2 extreme) were assigned a value corresponding to the rank of the traits in the carrier vs. non-carrier association results, treating deletion and duplication CNV carrier traits separately. We used a one-tailed Wilcoxon rank sum test to test whether the top 10% traits of each extreme category tend to have a shifted distribution for association with the (corresponding) carrier status (16p11.2 duplications and deletions for 16p11.2 extremes, 22q11.2 duplications and 22q11.2 deletions for 22q11.2 extremes).

### Significance threshold for association studies

The significance threshold used for each discovery MultiXcan or S-MultiXcan association study and conditional analysis was 0.05/(number of traits × number of CNV genes tested). In practice, this usually meant 5 traits and 127 CNV genes, for a threshold of *P* < 7.9 × 10^−5^. For replication studies, the significance threshold was set at 0.05 in order to test a single gene. The exception was in the BMI UK Biobank dataset. We first tried a phenotype-swapping approach to generate an expected distribution for the 16p11.2 genes. The distributions were null and did not yield meaningful comparisons. Instead, 100 random subsets of adjacent genes of approximately the same length and gene count as the CNV were tested for association with BMI. The 95th percentile of the MultiXcan *p* values for these genes was used as a permutation-based significance threshold.

In the gene-based PheWAS study, there were 1531 phecodes (each with at least 20 cases) tested overall, corresponding to a Bonferroni-corrected phenome-wide significance threshold of 3.3 × 10^−5^. For genes having no phenome-wide significant results, their top 15 associations, corresponding to the top 1% of the 1,531 phecodes, were used. In the carrier vs. non-carrier PheWAS, there were 1795 phecodes tested overall, corresponding to a Bonferroni-corrected phenome-wide significance threshold of 2.79 × 10^−5^. Additional traits meeting a false discovery rate threshold of 0.05 were considered in identifying traits both over-represented in carriers and represented in individual gene PheWAS.

### Graphical summary of selected PheWAS results

The *chordDiagram* method in the *circlize* package was used to generate the circle summary plots [81]. The gene-trait pairs we selected for Tables 1 and 2 were used as inputs, with the − log10 *p* value of association used as the weighting to determine the edge width. For the 22q11.2 circle plot, only associations with *P* < 5 × 10^−3^ were used in order to create a legible plot. Descriptions were cut off at 55 characters; to read the entire descriptions, see Tables 1 and 2.

**Table 1:** Selected 16p11.2 gene associations with PheWAS traits

Gene	PheWAS trait	*P* value	Reason for inclusion
NPIPB11	Psychosis^a^	1.04 × 10^−5^	Brain-related, PheWS
	Schizophrenia and other psychotic disorders	0.0016	Brain-related
	Dysphagia	0.0031	Del/dup
	Infantile cerebral palsy	0.0039	Dup, brain-related
BOLA2	Schizophrenia and other psychotic disorders	0.0082	Brain-related
	Psychosis^b^	0.0083	Brain-related
SLX1B	Psychosis^a^	3.03 × 10^−5^	Brain-related, PheWS
	Schizophrenia and other psychotic disorders	0.000606	Brain-related
CA5AP1	Developmental delays and disorders	0.005	Del/dup, brain-related
	Pervasive developmental disorders	0.01	Del/dup, brain-related
SPN	Failure to thrive (childhood)	0.0039	Dup
C16orf54	Essential hypertension^a^	2.8 × 10^−5^	PheWS
	Bariatric surgery	0.0019	Brain-related
PRRT2	Other specified nonpsychotic and/or transient mental disorders	0.0031	Brain-related
	Alteration of consciousness	0.0079	Brain-related
MVP	Dysphagia	0.003	Del/dup
	Symptoms involving head and neck	0.0073	Brain-related
CDIPT	GERD	0.0032	Del
SEZ6L2	Other specified nonpsychotic and/or transient mental disorders	0.0025	Brain-related
	Schizophrenia and other psychotic disorders	0.0029	Brain-related
	Alteration of consciousness	0.0029	Brain-related
ASPHD1	Substance addiction and disorders	0.0015	Brain-related
	Upper gastrointestinal congenital anomalies	0.0044	Del
KCTD13	Lack of coordination	0.0023	Del, brain-related
TMEM219	Mental retardation	0.00034	Del/dup, brain-related
TAOK2	Cardiomegaly	0.01	Dup
HIRIP3	Acute cystitis^a^	2.9 × 10^−6^	PheWS
	Disorders of uterus, NEC^a^	1.3 × 10^−5^	PheWS
INO80E	Skull and face fracture and other intercranial injury	1.9 × 10^−15^	Brain-related, PheWS
	Substance addiction and disorders	0.0032	Brain-related
	Other specified cardiac dysrhythmias	0.0034	Del
FAM57B	Upper gastrointestinal congenital anomalies	0.0011	Del
ALDOA	Neurological disorders	0.0014	Del/dup, brain-related
	Upper gastrointestinal congenital anomalies	0.0029	Del
	Antisocial/borderline personality disorder	0.0043	Brain-related
	Altered mental status	0.0043	Del, brain-related
	Other specified nonpsychotic and/or transient mental disorders	0.0052	Brain-related
	Abnormal movement	0.007	Del/dup, brain-related
	Convulsions	0.0072	Dup, brain-related
TBX6	Chromosomal anomalies and genetic disorders	0.0011	Del/dup
	Upper gastrointestinal congenital anomalies	0.0059	Del
YPEL3	Chromosomal anomalies and genetic disorders	0.0035	Del/dup
	Other specified cardiac dysrhythmias	0.0038	Del
	Delayed milestones	0.0053	Del/dup, brain-related
MAPK3	Substance addiction and disorders	0.00063	Brain-related
	Delayed milestones	0.0014	Del/dup, brain-related
	Aphasia/speech disturbance	0.0036	Del, brain-related
	Psychosis^b^	0.0054	Brain-related
	Upper gastrointestinal congenital anomalies	0.0092	Del
CORO1A	Dysphagia	0.00034	Del/dup
	Dementias	0.013	Brain-related
SULT1A3	Upper gastrointestinal congenital anomalies	0.0033	Del
	Obsessive-compulsive disorders	0.0042	Brain-related
	Altered mental status	0.006	Del, brain-related
	Swelling, mass, or lump in head and neck [Space-occupying lesion, intracranial NOS]	0.01	Brain-related
CD2BP2	Substance addiction and disorders	0.0034	Brain-related
	Dysphagia	0.0055	Del/dup
TBC1D10B	Schizophrenia and other psychotic disorders	0.0013	Drain-related
	Psychosis	0.0028	Brain-related
	Alcoholic liver damage	0.0045	Brain-related
	Lack of coordination	0.011	Del, brain-related
MYLPF	Morbid obesity	0.0037	Brain-related
ZNF48	Bariatric surgery^c^	0.0071	Brain-related
SEPT1	Other specified nonpsychotic and/or transient mental disorders	0.00055	Brain-related
	Alteration of consciousness	0.0018	Brain-related
	Ill-defined descriptions and complications of heart disease	0.0019	Dup
	Psychosis^c^	0.0035	Brain-related
	Substance addiction and disorders	0.0068	Brain-related

Possible reasons for inclusion are (1) del, dup, or del/dup: trait is over-represented in 16p11.2 deletion carriers, duplication carriers, or both (*P <* 2.8 × 10^−5^); (2) brain-related trait; (3) PheWS, phenome-wide significant

^a^Phenome-wide significant gene-trait pair (*P* < 3.3 × 10^−5^)

^b^Not significant after conditional analysis

^c^In an independent dataset, this brain-related gene-trait pair reached *P* < 0.05 and was in the top 5% of genes associated with this trait overall

**Table 2:** Selected 22q11.2 gene associations with PheWAS traits

Gene	PheWAS trait	*P* value	Reason for inclusion
TUBA8	Acute reaction to stress	0.0006	Brain-related
	Delirium dementia and amnestic and other cognitive disorders	0.0015	Brain-related
	Attention deficit hyperactivity disorder	0.0031	Brain-related
USP18	Aphasia	0.00066	Brain-related
	Pulmonary collapse; interstitial and compensatory emphysema	0.00091	Del
	Arrhythmia (cardiac) NOS	0.0026	Del
GGT3P	Endocrine and metabolic disturbances of fetus and newborn	0.00068	Del
	Respiratory failure	0.0015	Del
	Memory loss	0.016	Brain-related
DGCR6	Diseases of the larynx and vocal cords	0.0014	Del
	Tobacco use disorder	0.0086	Brain-related
PRODH	Gout and other crystal arthropathies^a^	1.3 × 10^−5^	PheWS
	Diseases of the larynx and vocal cords	0.005893	Del
	Voice disturbance	0.00801	Del
DGCR9	Gastrointestinal hemorrhage	0.00016	Del
TSSK1A	Hypoparathyroidism	0.0011	Del
	Disorders of parathyroid gland	0.0029	Del
SLC25A1	Acute upper respiratory infections of multiple or unspecified sites	0.00015	Del
CLTCL1	Anxiety, phobic and dissociative disorders	0.0054	Brain-related
C22orf39	Other disorders of tympanic membrane	0.0051	Del
	Abnormality of gait	0.0092	Dup, brain-related
CDC45	Hypoparathyroidism	0.00061	Del
	Impulse control disorder	0.0035	Brain-related
	Pervasive developmental disorders	0.011	Dup, brain-related
CLDN5	Eustachian tube disorders	0.0078	Del
TBX1	Curvature of spine	0.00083	Del
	Agorophobia, social phobia, and panic disorder	0.0013	Brain-related
	Personality disorders	0.0043	Brain-related
GNB1L	Delirium dementia and amnestic and other cognitive disorders	0.0023	Brain-related
	Heart valve disorders	0.0029	Del
	Dementias	0.0047	Brain-related
	Acute upper respiratory infections of multiple or unspecified sites	0.0071	Del
	Tachycardia NOS	0.0074	Del
ARVCF	Obsessive-compulsive disorders	0.0024	Brain-related
	Diseases of the larynx and vocal cords	0.0041	Del
	Chromosomal anomalies	0.0075	Del/dup
	Hypoparathyroidism	0.0094	Del
TANGO2	Autism	0.0011	Dup, brain-related
	Tension headache	0.002	Drain-related
	Antisocial/borderline personality disorder	0.0028	Drain-related
	Epilepsy, recurrent seizures, convulsions	0.0049	Del/dup, brain-related
DGCR8	Dependence on respirator [Ventilator] or supplemental oxygen	0.00059	Del
	Hallucinations	0.0061	Brain-related
TRMT2A	Other specified nonpsychotic and/or transient mental disorders	0.0033	Brain-related
	Alteration of consciousness	0.0061	Brain-related
RANBP1	Bariatric surgery	0.00034	Brain-related
	Obsessive-compulsive disorders	0.0011	Brain-related
	Pulmonary insufficiency or respiratory failure following trauma and surgery	0.0026	Del
	Acute upper respiratory infections of multiple or unspecified sites	0.0035	Del
ZDHHC8	Autism	0.0013	Dup, brain-related
	Tension headache	0.0035	Brain-related
	Acute reaction to stress	0.0049	Brain-related
RTN4R	Heart valve disorders	0.0035	Del
	Swelling, mass, or lump in head and neck [Space-occupying lesion, intracranial NOS]	0.0044	Brain-related
	Tension headache	0.0065	Brain-related
	Epilepsy, recurrent seizures, convulsions	0.0084	Del/dup, brain-related
DGCR6L	Disorders of fluid, electrolyte, and acid-base balance	0.0065	Del
	Other persistent mental disorders due to conditions classified elsewhere	0.0077	Brain-related
USP41	Impacted cerumen	0.0026	Del
	Esophagitis, GERD and related diseases	0.006	Del
	Alzheimer's disease	0.0072	Brain-related
ZNF74	Septicemia	0.00061	Del
	Mood disorders	0.0053	Brain-related
	Heart valve disorders	0.0057	Del
SCARF2	Mood disorders^a, c^	1.3 × 10^−5^	Brain-related, PheWS
	Depression	0.00014	Brain-related
	Schizophrenia	0.00027	Brain-related
	Blood in stool	0.00071	Del
	Obsessive-compulsive disorders	0.001	Brain-related
	Alteration of consciousness	0.0011	Brain-related
	Schizophrenia and other psychotic disorders	0.003	Brain-related
	Major depressive disorder	0.0033	Brain-related
	Respiratory conditions of fetus and newborn	0.0035	Del
KLHL22	Premature beats	0.00013	Del
	Valvular heart disease/ heart chambers	0.0051	Del
	Overweight, obesity and other hyperalimentation	0.0064	Brain-related
	Mood disorders	0.01	Brain-related
	Heart transplant/surgery	0.011	Del
	Posttraumatic stress disorder	0.012	Brain-related
	Obsessive-compulsive disorders	0.012	Brain-related
KRT18P5	Acute posthemorrhagic anemia	0.00048	Del
	Other persistent mental disorders due to conditions classified elsewhere	0.0016	Brain-related
MED15	Other upper respiratory disease	0.0019	Del
	Mood disorders	0.0120	Brain-related
SMPD4P1	Other disorders of intestine	0.001	Del
	Acidosis	0.0039	Del
	Acid-base balance disorder	0.0054	Del
	Renal failure	0.0059	Del
POM121L4P	Acute reaction to stress	0.0022	Brain-related
	Convulsions	0.0072	Del, brain-related
PI4KA	Disorders of iris and ciliary body^a^	1.1 × 10^−7^	PheWS
	Muscular calcification and ossification^a^	7.3 × 10^−6^	PheWS
	Disorders resulting from impaired renal function^a^	2.2 × 10^−5^	PheWS
	Stricture/obstruction of ureter^a^	3.1 × 10^−5^	PheWS
	Disorders of calcium/phosphorus metabolism	5.7 × 10^−5^	Del
	Renal failure	0.0007	Del
SERPIND1	Other anemias	0.00044	Del
	Essential hypertension	0.00045	Del
	Renal failure	0.0009	Del
	Acidosis	0.001	Del
	Septicemia	0.0011	Del
SNAP29	Curvature of spine	0.0015	Del
	Morbid obesity	0.0045	Brain-related
AIFM3	Renal failure^a^	2.3 × 10^−5^	Del, PheWS
	Pulmonary collapse; interstitial and compensatory emphysema	0.0053	Del
	Mood disorders^c^	0.006	Brain-related
LZTR1	Malignant neoplasm, other^a^	1.4 × 10^−5^	PheWS
	Renal failure	0.00077	Del
	Septicemia	0.0014	Del
	Obsessive-compulsive disorders	0.0018	Brain-related
	Esophagitis, GERD and related diseases	0.0054	Del
	Pulmonary collapse; interstitial and compensatory emphysema	0.0056	Del
TUBA3FP	Psychogenic disorder	0.0017	Brain-related
	Hypothyroidism NOS	0.0074	Del
P2RX6	Morbid obesity	0.00012	Brain-related
	Other perinatal conditions of fetus or newborn	0.00022	Del
	Renal failure	0.00067	Del
	Eating disorder	0.0065	Brain-related
P2RX6P	Morbid obesity^b^	0.00043	Brain-related
	Paroxysmal tachycardia, unspecified	0.0014	Del
	Eating disorder	0.0072	Brain-related
BCRP2	Disorders of parathyroid gland	0.0078	Del
GGT2	Depression	0.0038	Brain-related
	Hypovolemia	0.0043	Del
	Chromosomal anomalies and genetic disorders	0.0059	Del/dup
	Mood disorders	0.0064	Brain-related
POM121L8P	Immunity deficiency	0.0063	Del
HIC2	Bacterial infection NOS	0.00023	Del
	Mood disorders^c^	0.000464	Brain-related
	Tension headache	0.00069	Brain-related
	Swelling, mass, or lump in head and neck [Space-occupying lesion, intracranial NOS]	0.00091	Brain-related
	Esophagitis, GERD and related diseases	0.002	Del
	Pleurisy; pleural effusion	0.0023	Del
	Posttraumatic stress disorder	0.0028	Brain-related
	Pervasive developmental disorders	0.0031	Dup, brain-related
TMEM191C	Other CNS infection and poliomyelitis^a^	7.2 × 10^−6^	PheWS
	Eustachian tube disorders	0.0022	Del
	Renal failure	0.0029	Del
	Septicemia	0.0038	Del
	Bacteremia	0.0073	Del
	Diseases of hard tissues of teeth	0.008431	Del
RIMBP3C	Cellulitis and abscess of oral soft tissues^a^	1.8 × 10^−5^	PheWS
	Pulmonary insufficiency or respiratory failure following trauma and surgery	0.00047	Del
	Obsessive-compulsive disorders	0.0018	Brain-related
UBE2L3	Acute reaction to stress	0.0019	Brain-related
YDJC	Swelling, mass, or lump in head and neck [Space-occupying lesion, intracranial NOS]	0.00025	Brain-related
	Symptoms involving head and neck	0.00072	Brain-related
	Ill-defined descriptions and complications of heart disease	0.0027	Del
	Speech and language disorder	0.0042	Del, brain-related
CCDC116	Abdominal aortic aneurysm^a^	1.9 × 10^−6^	PheWS
	Respiratory conditions of fetus and newborn	0.0032	Del
PPIL2	Arrhythmia (cardiac) NOS	0.006	Del

Possible reasons for inclusion are (1) del, dup, or del/dup: trait is over-represented in 16p11.2 deletion carriers, duplication carriers, or both (*P <* 2.8 × 10^−5^); (2) brain-related trait; (3) PheWS, phenome-wide significant

^a^Phenome-wide significant gene-trait pair (*P* < 3.3 × 10^−5^)

^b^Not significant after conditional analysis

^c^In an independent dataset, this brain-related gene-trait pair reached *P* < 0.05 and was in the top 5% of genes associated with this trait overall

## Results

### Individual genes at 16p11.2 are associated with schizophrenia, IQ, and BMI

In order to find genes at copy number variant loci driving brain-related disorders, we performed an association analysis between imputed gene expression levels and five traits: schizophrenia, IQ, BMI, bipolar disorder, and ASD. It has been observed that copy number variants (including 16p11.2 and 22q11.2) affect expression of nearby genes [44, 45, 82]. As flanking genes affected by copy number variation may be relevant to phenotype, we additionally considered genes 200 kb in each direction from each CNV [83]. Overall, we tested 52 coding and 30 noncoding genes at or near 22q11.2 and 37 coding and 8 noncoding genes at or near 16p11.2 for which a predictive model was available (Additional file 2: Table S2, Additional file 3: Fig. S1). As cis-eQTLs are often shared among tissues, we pooled together information from all tissues in GTEx to boost our power to detect brain-related traits [58].

**Fig. 2:**
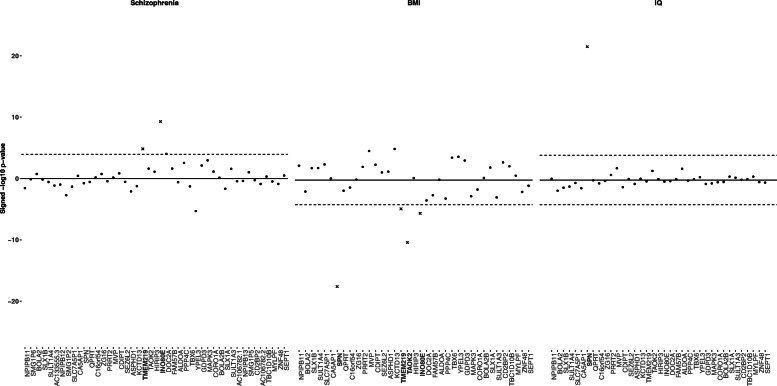
Association between 16p11.2 genes and three brain-related traits. Association between predicted expression of 16p11.2 genes and schizophrenia (left), BMI (middle), IQ (right) using MultiXcan (schizophrenia) and S-MultiXcan (BMI, IQ). Genes are listed on the horizontal access in order of chromosomal position. The − log10 *p* values on the vertical axis are given a positive or negative direction based on the average direction of the single-tissue results. The significance threshold, *P* < 7.9 × 10^−5^, is a Bonferroni correction on the total number of 16p11.2 and 22q11.2 genes (127) tested across 5 traits (0.05/(5 × 127)). Genes exceeding the significance threshold in the expected direction (positive for schizophrenia, negative for BMI, either for IQ) are denoted as x’s

Two genes at 16p11.2 show predicted expression positively associated (*P* < 7.9 × 10^−5^) with schizophrenia (Fig. 2; Additional file 6: Table S5): *TMEM219* (*P* = 1.5 × 10^−5^) and *INO80E* (*P* = 5.3 × 10^−10^). This positive direction of effect is consistent with the association between 16p11.2 duplications and schizophrenia [2]. An additional gene, *YPEL3*, was significantly associated with schizophrenia in the negative direction (*P* = 4.9 × 10^−6^). For IQ, there was one strong positive association at the 16p11.2 locus (Fig. 2; Additional file 6: Table S5): *SPN* (*P* = 2.9 × 10^−22^). Intellectual disability is observed in both deletions and duplications of 16p11.2, so there was no expected direction of effect [3, 14]. Four genes showed negative association with BMI (Fig. 2; Additional file 6: Table S5): *SPN* (*P* = 6.2 × 10^−18^), *TMEM219* (*P* = 2.2 × 10^−5^), *TAOK2* (*P* = 8.5 × 10^−11^), and *INO80E* (*P* = 1.0 × 10^−7^). We focused on genes with negative associations with BMI because, in humans, obesity is associated with deletions at 16p11.2 [1, 16]. Two additional genes, *KCTD13* (*P* = 9.5 × 10^−6^) and *MVP* (*P* = 2.1 × 10^−5^), were significantly associated with BMI in the positive direction. No gene at 16p11.2 was significantly associated with bipolar disorder or ASD (Additional file 6: Table S5, Additional file 3: Fig. S3). No individual genes at or near 22q11.2 had predicted expression significantly associated with any of the five traits (Additional file 6: Table S5, Additional File 3: Fig. S4).

### Follow-up conditional analyses narrow down genes driving schizophrenia and BMI

To replicate our analysis, we used a large cohort from the UK Biobank for which GWAS summary statistics were available for multiple brain-related traits (Additional file 1: Table S1) [66]. The predicted expression of *INO80E* and *TMEM219* from the discovery analyses were associated (*P* < 0.05) with having an ICD10 diagnosis of schizophrenia (ICD10: F20, 198 cases: *INO80E P* = 0.04, *TMEM219 P =* 0.03, Additional file 7: Table S6). Although this is only nominally significant, it is notable that these genes are in the 3rd percentile of schizophrenia associations genome-wide within UK Biobank.

The UK Biobank GWAS of BMI is highly inflated, including in the 16p11.2 region. Nearly every 16p11.2 gene showed association at the previously used threshold (*P* < 7.9 × 10^−5^). Using a permutation-based approach within individual-level data, we adjusted the significance threshold to 8.8 × 10^−11^. All genes from the discovery analysis replicated (Additional file 7: Table S6): *SPN* (*P* = 6.1 × 10^−23^), *KCTD13* (*P =* 1.2 × 10^−30^), *TMEM219* (*P =* 7.1 × 10^−37^), *MVP* (*P =* 5.1 × 10^−11^), and *INO80E (P* = 1.9 × 10^−27^). We were not able to replicate the IQ result in the UK Biobank, because the UK Biobank sample overlapped with our discovery GWAS.

We performed an additional fine-mapping study on the three genes associated with schizophrenia. Linkage disequilibrium between the eQTL SNPs in predictive models may lead to correlation among predicted expressions for nearby genes, so it is possible that not all three detected association signals are independent. The predicted expressions of *INO80E*, *YPEL3*, and *TMEM219* were moderately correlated (the correlation of *INO80E* with the other genes is in the range of − 0.4 to 0.37 across GTEx tissues, for example), consistent with the relationships between the observed expressions of these genes (measured expression of *INO80E* is correlated with measured expression of the other genes in the range − 0.36 to 0.31). In order to pick out the gene(s) driving the association signal, we used a conditional analysis approach (Additional file 8: Table S7). We observed that after adjusting the predicted expression of the other CNV genes for the predicted expression of *INO80E*, no gene was significantly associated with schizophrenia. However, when we adjusted the predicted expression of *INO80E* by the predicted expressions of the other two highly associated genes, *INO80E* remained significantly associated with schizophrenia (*P* = 2.3 × 10^−6^). The same pattern was not observed for *TMEM219* or *YPEL3*, suggesting *INO80E* explains the entire 16p11.2 signal for schizophrenia.

While we did not have individual-level data for the GIANT consortium, we obtained individual-level BMI data from the UK Biobank [68]. We performed an analogous conditional analysis on the six genes associated with BMI, *SPN*, *INO80E*, *TMEM219*, *TAOK2* in the negative direction, as well as *KCTD13* and *MVP* in the positive direction. Due to the inflation in the UK Biobank data, all these genes had very low *p* values even after conditioning; however, we see that some genes’ association results stayed in the same range, while others increased in *p* value by five orders of magnitude or more after adjusting by the other five genes. Based on these observations, it is likely that *SPN* (*P*_*UKBB*_ *=* 6.1 × 10^−23^, *P*_*cond*_ *=* 7.5 × 10^−21^), *INO80E* (*P*_*UKBB*_ = 1.9 × 10^−27^, *P*_*cond*_ *=* 2.8 × 10^−32^), and *KCTD13* (*P*_*UKBB*_ *=* 1.2 × 10^−30^, *P*_*cond*_ *=* 4 × 10^−27^) were independently associated with BMI, while *TMEM219* (*P*_*UKBB*_ *=* 7 × 10^−37^, *P*_*cond*_ *=* 2.3 × 10^−18^), *TAOK2* (*P*_*UKBB*_ *=* 4.2 × 10^−29^, *P*_*cond*_ *=* 2.3 × 10^−19^), and *MVP* (*P*_*UKBB*_ *=* 5.1 × 10^−11^, *P*_*cond*_ = 5 × 10^−6^) were significant in the discovery analysis primarily due to correlation with one of the independent genes.

To validate that our approach explained all GWAS signal at the locus, we took two phenotypes in which we had both GWAS signal and individual-level data available—PGC Schizophrenia and UK Biobank BMI—and conditioned the MultiXcan analysis on lead GWAS SNP(s) in those datasets that were also eQTLs. In schizophrenia (where *INO80E* is our proposed sole driver gene), conditioning on one GWAS SNP (rs4788200, GWAS *P =* 2.8 × 10^−10^) was sufficient to explain the GWAS peak in the region (Additional file 3: Fig. S2). Conditioning the MultiXcan analysis on this SNP successfully removed all association signals, including for *INO80E* (Additional file 3: Fig. S2). In BMI (where we propose three independent genes, *INO80E*, *KCTD13*, *SPN*), conditioning on four GWAS/eQTL SNPs was sufficient to explain both the GWAS and MultiXcan signal (Additional file 3: Fig. S2). These were rs4787491 (GWAS *P* = 7.6 × 10^−17^), rs9936474 (GWAS *P* = 5.1 × 10^−31^), rs2008514 (GWAS *P* = 3.3 × 10^−29^), and rs8046707 (GWAS *P* = 3.2 × 10^−19^). The first two SNPs explain the GWAS signal within the region, and the latter two come from more distal GWAS peaks that are nevertheless involved in the expression prediction of 16p11.2 genes; as a result, four SNPs are needed to fully nullify the MultiXcan signal. The schizophrenia variant rs4788200 is not a strong eQTL for any gene-tissue pair, but it appears in the models for *INO80E* in 22/37 tissues where *INO80E* has models. Similarly, one of the BMI SNPs, rs4787491 is an expression-decreasing eQTL for *INO80E* in 35/37 tissues and is generally strong: the distribution of weights of this SNP was significantly different from the distribution of all *INO80E*-predicting SNPs, (*P =* 4.8 × 10^−13^, Kolmogorov-Smirnov test). We conclude that our approach is sufficient for explaining GWAS signal and that the multi-SNP predictive models involving both nearby and more distal SNPs are advantageous.

### Phenome-wide association studies identify previously known and novel traits associated with 16p11.2 and 22q11.2 carrier status

**Fig. 3:**
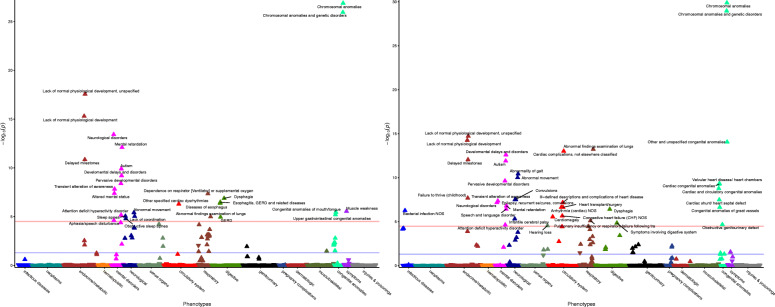
Clinical traits over-represented in 16p11.2 deletion and duplication carriers. CNV carriers were identified in the EHR by keyword search and chart review (left, 16p11.2 deletions [*n* = 48], right, 16p11.2 duplications [*n* = 48], see “Methods”). Controls included all individuals without the CNV within the medical home population at Vanderbilt (*n* ~ 707,000). The *x*-axis represents the PheWAS codes that are mapped from ICD-9/ICD-10 codes, grouped and color-coded by organ system. The *y*-axis represents the level of significance (− log_10_*p*). The horizontal red line indicates a Bonferroni correction for the number of phenotypes tested in this PheWAS (*p* = 0.05/1,795 = 2.8 × 10^−5^); the horizontal blue line indicates *p* = 0.05. Each triangle represents a phenotype. Triangles represent direction of effect; upward pointing arrows indicate phenotypes more common in cases. Covariates included age, sex, and self-reported race extracted from the EHR. Phenotypes reaching Bonferroni-corrected significance level are labeled in plot

**Fig. 4:**
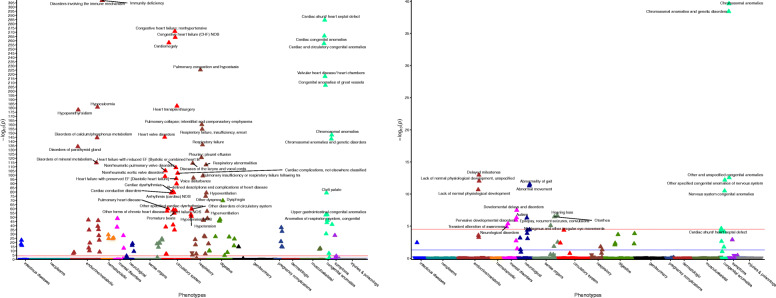
Clinical traits over-represented in 22q11.2 deletion and duplication carriers. CNV carriers were identified in the EHR by keyword search and chart review (left, 22q11.2 deletions [*n* = 388], right, 22q11.2 duplications [*n* = 43], see “Methods”). Controls included all individuals without the CNV within the medical home population at Vanderbilt (*n* ~ 707,000). The *x*-axis represents the PheWAS codes that are mapped from ICD-9/ICD-10 codes, grouped and color-coded by organ system. The *y*-axis represents the level of significance (− log_10_*p*). The horizontal red line indicates a Bonferroni correction for the number of phenotypes tested in this PheWAS (*p* = 0.05/1,795 = 2.8 × 10^−5^); the horizontal blue line indicates *p* = 0.05. Each triangle represents a phenotype. Triangles represent direction of effect; upward pointing arrows indicate phenotypes more common in cases. Covariates included age, sex, and self-reported race extracted from the EHR. Top phenotypes (*P* < 1.0 × 10^−50^) are labeled in the 22q11.2 deletion plot (left). Phenotypes reaching Bonferroni-corrected significance level are labeled in the 22q11.2 duplication plot (right)

While GWAS datasets provide insight into the impact of genes on ascertained brain-related traits, the 16p11.2 and 22q11.2 CNVs may contribute to a wide spectrum of traits, including milder manifestations of brain-related traits. Thus, biobanks containing both genetic and clinical data can tell us about broader clinical impacts on medical traits. We queried the de-identified electronic health records for 3.1 million patients at VUMC to explore the impacts of the 16p11.2 and 22q11.2 CNVs, as well as their individual genes, on the medical phenome in a representative population [64]. CNV diagnoses are documented in the medical records, which led us to ask: what are the specific clinical phenotypes that are common in individuals identified as 16p11.2 or 22q11.2 CNV carriers? Carriers were identified by diagnosis of 16p11.2 or 22q11.2 deletion/duplication (or syndromic names for 22q11.2, see methods) in their medical record, and over 700,000 individuals were used as controls. We performed a phenome-wide association study (PheWAS) between 16p11.2 and 22q11.2 deletion/duplication carriers and controls against 1795 medical phenotype codes (Figs. 3 and 4) [77, 80]. Traits that were significantly over-represented in carriers (*P <* 2.8 × 10^−5^) fell into three major categories: (1) known primary CNV clinical features, including possible reasons for the referral of the patient for genetic testing (i.e., neurodevelopmental concerns, epilepsy, congenital heart defects), (2) secondary CNV features known to be present in carriers but unlikely to be a primary reason for referral for genetic testing, (3) novel diagnoses not previously reported (Fig. 3, Fig. 4, Additional file 9: Table S8). We chose to focus on traits present in at least 5% of carriers to avoid over-interpreting rare traits.

16p11.2 deletion carrier status was associated with developmental diagnoses (Fig. 3): *lack of normal physiological development* (*P* = 2.8 × 10^−18^), *developmental delays and disorders* (*P* = 6.3 × 10^−10^), *delayed milestones* (*P* = 1.4 × 10^−11^) [3]*.* In addition, 16p11.2 deletion carrier status was associated with *autism* (*P* = 1.3 × 10^−10^) and *mental retardation* (*P* = 7.9 × 10^−13^) [5]. The digestive diagnosis of *GERD* (*P =* 1.1 × 10^−5^) has been previously observed in carriers but was unlikely to be a primary reason for genetic testing [84]. *GERD* was accompanied by other digestive diagnoses such as *dysphagia* (*P* = 1.3 × 10^−7^) and *diseases of esophagus* (*P* = 4.3 × 10^−7^)*. Muscle weakness* (*P =* 2.8 × 10^−6^) and *abnormal movements* (*P =* 3.9 × 10^−6^) are consistent with neurological traits reported in 16p11.2 deletion carriers such as hypotonia and motor impairments [85]. *Sleep apnea* (*P =* 8.9 × 10^−6^) was a novel phenotype, potentially related to increased BMI in deletion carriers.

16p11.2 duplication carrier status was similarly associated with developmental diagnoses (Fig. 3): *lack of normal physiological development* (*P* = 5.6 × 10^−15^), *developmental delays and disorders* (*P* = 2.5 × 10^−13^), *delayed milestones* (*P* = 9.0 × 10^−13^), *autism* (*P* = 1.3 × 10^−12^), and *mental retardation* (*P* = 1.6 × 10^−7^) [3, 5]. 16p11.2 duplication carriers status was also associated with multiple heart defects, including *valvular heart disease/heart chambers* (*P =* 4.6 × 10^−10^) and *cardiac shunt/heart septal defect* (*P* = 3.2 × 10^−8^), both of which have been reported previously [86]. 16p11.2 duplications are known to be a risk factor for epilepsy and were associated with an epilepsy-related diagnosis of *convulsions* (*P* = 2.9 × 10^−8^) in the biobank [3, 87]. *Infantile cerebral palsy* (*P* = 4.9 × 10^−6^), while a potential reason for genetic testing, has not previously been associated with 16p11.2 duplications. While the 16p11.2 CNV contains genes such as *SPN* and *MVP* that are active in the immune system, there is no prior evidence of the susceptibility of duplication carriers to infection, making the diagnosis *Bacterial infection NOS* (*P* = 5.5 × 10^−7^) a novel finding.

For 22q11.2 deletion carriers, the canonical associated features were cardiac defects such as *cardiomegaly* (*P* = 3.5 × 10^−258^) and *cardiac shunt/heart septal defects* (*P* = 4.7 × 10^−285^) (Fig. 4) [6, 7]. Other highly associated diagnoses were developmental: *lack of normal physiological development* (*P* = 1.7 × 10^−47^), *developmental delays and disorders* (*P =* 6.3 × 10^−29^), *delayed milestones* (*P* = 6.0 × 10^−11^) [6, 7]. Congenital anomalies such as *cleft palate* (*P =* 9.4 × 10^−80^) were also over-represented. The secondary known traits for 22q11.2 deletion carriers included *immunity deficiency* (*P <* 10^−285^), and *disorders involving the immune mechanism* (*P* < 10^−285^). Previously, it has been reported that 50% of 22q11.2 deletion carriers have T cell dysfunction and 17% have humoral dysfunction [7]. Very few traits over-represented in 22q11.2 deletion carriers were novel; one of these was *hyperpotassemia* (*P* = 1.4 × 10^−10^).

22q11.2 duplication carrier status was also associated with developmental diagnoses (Fig. 4): *delayed milestones* (*P* = 1.1 × 10^−13^), *lack of normal physiological development* (*P* = 9.7 × 10^−13^), *pervasive developmental disorders* (*P* = 1.2 × 10^−6^) [8]*.* 22q11.2 duplication status was associated with cardiac phenotypes such as *cardiac shunt/ heart septal defect* (*P =* 2.3 × 10^−5^). Cardiac features have not as often been reported in 22q11.2 duplication carriers compared to 22q11.2 deletion carriers [8]. Remaining traits such as *abnormality of gait* (*P =* 3.1 × 10^−12^) and *hearing loss* (*P =* 2.1 × 10^−7^) have also been seen in 22q11.2, including as indications for genetic testing [88].

### Phenome-wide association studies identify phenotypic consequences of expression variation in 16p11.2 and 22q11.2 genes

As our study of the impact of the entire CNV on phenotype confirmed our ability to detect important CNV-associated traits within the BioVU biobank, our next goal was to catalog how each individual CNV gene might affect the medical phenome. We generated predicted expression for CNV and flanking genes, as in the initial GWAS analyses, for the 48,630 non-CNV carrier individuals genotyped in BioVU. We tested 1531 medical phenotypic codes meeting frequency criteria (*n* = 20 cases) in this subset. There were six phenome-wide significant (*P* < 3.3 × 10^−5^) gene-trait associations at 16p11.2 including the following: *INO80E* with *skull and face fracture and other intercranial injury* (*P* = 1.9 × 10^−15^), *NPIPB11* with *psychosis* (*P* = 1.0 × 10^−5^), and *SLX1B* with *psychosis* (*P* = 3.0 × 10^−5^). There were eleven phenome-wide significant gene-trait associations at 22q11.2 including as follows: *AIFM3* with *renal failure (P =* 2.3 × 10^−5^), *LZTR1* with *malignant neoplasm*, *other* (*P =* 1.4 × 10^−5^), *SCARF2* with *mood disorders* (*P =* 1.3 × 10^−5^), *PI4KA* with *disorders of iris and ciliary body* (*P* = 1.1 × 10^−7^), and *disorders resulting from impaired renal function* (*P* = 2.2 × 10^−5^)*.* These include two renal traits, consistent with the 22q11.2 deletion carrier status association with *renal failure.* The associations of *LZTR1* and *PI4KA* with neoplasms and eye disorders correspond to similar traits associated with these genes in prior literature [89–91].

Previously established gene-trait associations came up as suggestive (top 1 percentile), although not phenome-wide significant, associations in the BioVU cohort. *TBX1*, a gene at 22q11.2 tied to heart development, had *other chronic ischemic heart disease*, *unspecified* (*P =* 0.001), *endocarditis* (*P =* 0.0046), *cardiomyopathy* (*P =* 0.0055), and *coronary atherosclerosis* (*P =* 0.0076) among its top 1% phenome associations [28–31]. *TBX6* at 16p11.2, which has a role in bone development and scoliosis, has *pathologic fracture of vertebrae* in its top 1% phenome associations (*P =* 0.0028) [92–94]. *TANGO2* mutations at 22q11.2 have been associated with metabolic abnormalities such as hypoglycemia, as well as epilepsy, and our PheWAS for *TANGO2* showed *abnormal glucose* (*P =* 0.0013) and *epilepsy*, *recurrent seizures*, *and convulsions* (*P =* 0.0049) as top phenotypes [95, 96]. We identified additional genes at 16p11.2 and 22q11.2 that are associated with Mendelian traits, using OMIM [79], and browsed our PheWAS for potentially similar clinical traits, including those not meeting the top 1 percentile threshold. We find that of 13 such genes, 7 have a relevant clinical trait at *P* < 0.05, and 12 at *P* < 0.1. In 6 of the 13 genes, the relevant clinical traits are within the top 1% of PheWAS associations for the gene (Additional file 4: Table S3).

As few gene-trait pairs reached phenome-wide significance and established associations were present at more nominal levels, we also considered traits that did not meet the significance threshold in our analysis but were in the top 1% of phenotypic associations for a given gene (Additional file 10: Table S9). We found that traits categorized as “mental disorders” were over-represented in the top 1% of the phenome of CNV genes (*P* = 5.2 × 10^−5^). Of all 17 clinical categories tested, “mental disorders” was the only category with enrichment *p* value meeting multiple testing thresholds (Additional file 11: Table S10). This suggested that the effect of CNV genes is more widespread on brain-related traits than simply those detected as statistically significant.

**Fig. 5:**
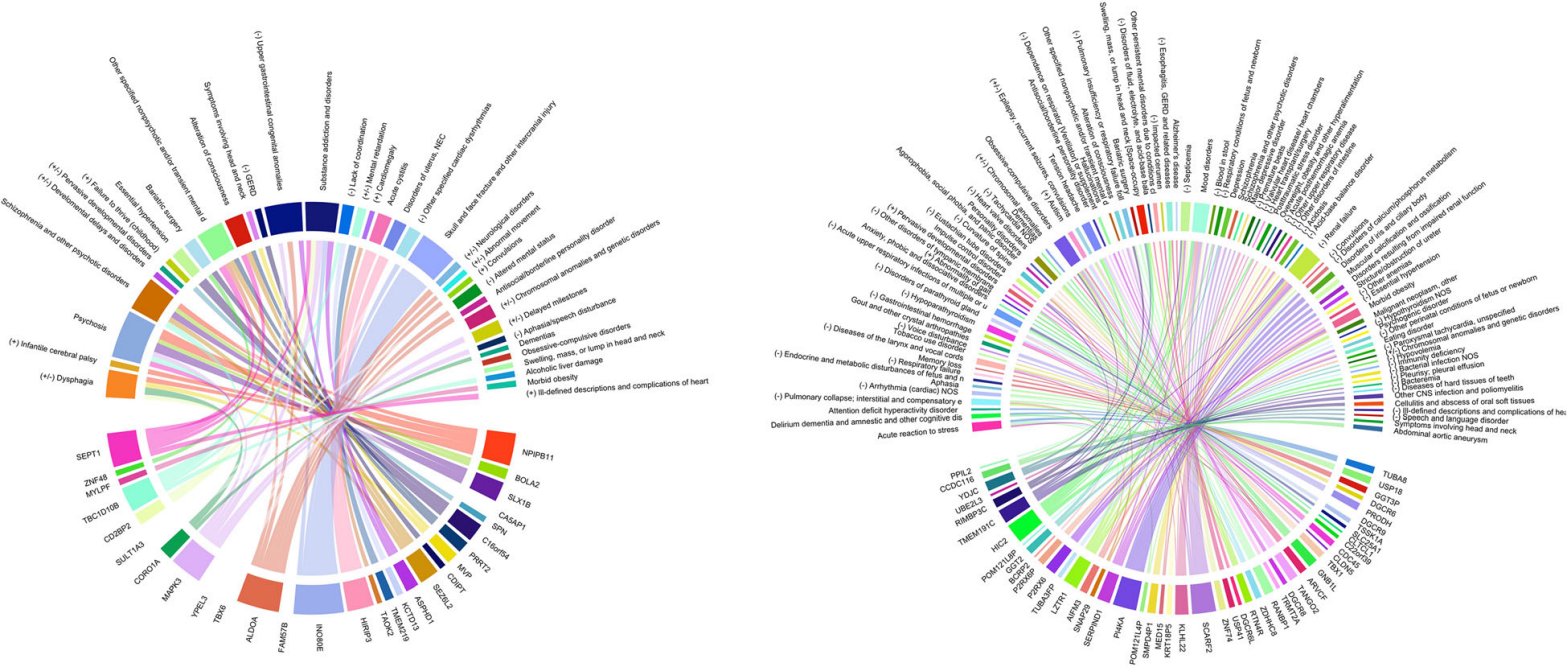
Graphical summary of selected PheWAS results by gene. Each circle contains the CNV genes, in chromosomal order, on the bottom, and their associated PheWAS traits at the top. Genes are connected to their PheWAS-associated traits, with the width of the line proportional to the − log10 *p* value of the association. If a trait is also over-represented in duplication and/or deletion carriers, it is marked with a + (duplications), − (deletions), or +/− (both). The complete list of gene-trait pairs can be found in Tables 1 and 2, and Supplemental Table S9 in Additional file 10

Some of the top 1% PheWAS traits for CNV genes overlapped with the original five traits we studied: schizophrenia, IQ, BMI, bipolar disorder, and ASD. At 16p11.2, there were genes whose top PheWAS results included schizophrenia-related traits (*psychosis*, *schizophrenia and other psychotic disorders*), IQ-related traits (*developmental delays and disorders*, *mental retardation*, *delayed milestones*), BMI-related traits (*bariatric surgery*, *morbid obesity*), and ASD-related traits (*pervasive developmental disorders*) (Table 1, Fig. 5). At 22q11.2, there were genes whose top PheWAS results included schizophrenia-related traits (*hallucinations*), BMI-related traits (*overweight*, *obesity and other hyperalimentation*, *morbid obesity*), ASD-related traits (*autism*, *speech and language disorder*), and bipolar-related traits (*mood disorders*) (Table 2, Fig. 5). We could not perform strict independent replication for these associations because many of these traits are difficult to define in the same way across datasets (for example *Speech and language disorder* vs. *Autism*). Instead, we compared the top association statistics within our GWAS discovery and replication datasets for the genes identified to be associated with brain-related traits in PheWAS as an extension of this study (Additional file 8: Table S7). The following genes were associated at *P* < 0.05 and also in the top 5th percentile within at least one of the GWAS discovery or replication datasets (Additional file 7: Table S6): *SEPT1* (*psychosis*—in UK Biobank schizophrenia 20002_1289 *P* = 0.03), *AIFM3* (*mood disorders*—in UK Biobank bipolar F31 *P =* 0.04), *SCARF2* (*mood disorders*—in UK Biobank bipolar F31 *P* = 0.003), *HIC2* (*mood disorders*—in UK Biobank bipolar 20002_1991, *P =* 0.004), *ZNF48* (*bariatric surgery*—in UK Biobank BMI 3.7 × 10^−6^). Of these, the association between *SCARF2* and *mood disorders* reached phenome-wide significance in the PheWAS.

Predicted expression may be correlated between nearby genes, thus multiple genes can share a PheWAS trait association due to correlation alone. We are underpowered for independence testing for the majority of our GWAS traits, but we selected several notable traits that appeared in multiple genes to test for independence, in the same way as in our GWAS analysis (Additional file 8: Table S7). We performed a conditional analysis on 16p11.2 genes whose top phenome associations included *psychosis: NPIPB11*, *BOLA2*, *MAPK3*, *SEPT1*, *SLX1B*, *TBC1D10B.* By comparing whether the *p* value of association stayed constant vs. increased after conditioning, we found that *NPIPB11*, *SEPT1*, *SLX1B*, and *TBC1D10B* were likely independent associations, whereas *BOLA2* and *MAPK3* may be associated with *psychosis* at least partly by correlation with the other four. We also performed the same analysis for 22q11.2 genes whose top phenome associations included *morbid obesity*: *SNAP29*, *P2RX6*, *P2RX6P.* Of these genes, the only one with a *p* value increase was *P2RX6P*, suggesting that its association with *morbid obesity* may be explained at least in part by another gene. From conditional analysis, we see evidence of a multigenic contribution to both traits from CNV genes.

### Genes in 16p11.2 and 22q11.2 are associated with traits that are also over-represented in carriers

We originally hypothesized that small variations in CNV gene expression would be associated with phenotypes resembling those that were present in CNV carriers, perhaps with smaller effects. Our use of electronic health records first on the entire CNV itself, then on individual genes allows us to detect these potential effects across traits. Unlike the five brain-related traits that we originally chose, many of the traits in the EHRs do not have similar large GWAS datasets available. Considering that our non-ascertained biobank is not well-powered for less common traits, we chose to focus on the top one percentile of the phenome associations rather than the few associations that passed the phenome-wide significance threshold.

Traits that were found both in 16p11.2 carriers and in individual genes’ PheWAS results included primary CNV traits such as *mental retardation* and *delayed milestones*, as well as secondary traits such as *dysphagia* and *convulsions* (Table 1, Fig. 5). There were six genes (*ASPHD1*, *FAM57B*, *ALDOA*, *TBX6*, *MAPK3*, *SULT1A3)* whose top PheWAS associations included the 16p11.2 deletion-associated trait of *upper gastrointestinal congenital anomalies*, though we are underpowered to know whether all these signals are independent. Of the genes that we found as drivers in the first analysis of GWAS datasets, we note that *INO80E*’s top PheWAS results overlap the 16p11.2 deletion-associated trait *other specified cardiac dysrhythmias* and *SPN*’s top PheWAS results overlap the 16p11.2 duplication-associated trait of *failure to thrive (childhood*).

Over 30 genes at 22q11.2 had a top PheWAS trait overlapping a trait over-represented in 22q11.2 duplication or deletion carriers (Table 2, Fig. 5). Top PheWAS results for 22q11.2 genes included primary cardiac traits such as *tachycardia* (*P2RX6P*, *GNB1L*) and primary brain-related traits such as *autism* (*TANGO2*, *ZDHHC8).* We also found genes with top PheWAS results overlapping secondary traits from the carrier screen, such as *diseases of the larynx and vocal cords* (*DGCR6*, *PRODH*, *ARVCF*).

It is difficult to meaningfully compare the carrier screen to the gene-based PheWAS results because the effects of modest expression variation in an individual gene are not necessarily expected to be the same as those of the deletion or duplication of an entire locus. We tested whether the top associations from individual gene PheWAS results were enriched for EHR phenotypes over-represented in carriers. We did this by analyzing where top PheWAS traits associated with CNV genes were ranked within PheWAS results of carrier status. We found no evidence for enrichment in 16p11.2 duplications, 16p11.2 deletions, 22q11.2 duplications, or 22q11.2 deletions (Additional file 3: Fig. S3). As an alternate way to compare the two PheWAS approaches by ‘mimicking’ the CNV effects, we identified individuals in the genotyped cohort in BioVU that had the most extreme (2nd percentile) predicted expression across CNV genes in a region and were thus the most similar we could identify to true CNV carriers (see “Methods”). The top 10% of traits over-represented in this “extreme expression non-carrier” group were examined for their distribution within ranked (by *p* value) lists of traits in CNV carriers. We found that in all four cases (16p11.2 deletions, 16p11.2 duplications, 22q11.2 deletions, 22q11.2 duplications), the top traits in the “extreme expression non-carrier” group were more likely to rank near the top of the CNV carrier traits than would be expected by chance; the distribution was significantly shifted for 22q11.2 genes (*P* = 8.9 × 10^−15^, mean rank 487/1795, 22q11.2 deletions; *P* = 6.1 × 10^−8^, mean rank 563/1784, 22q11.2 duplications; *P =* 0.18, mean rank 770/1816, 16p11.2 deletions; *P =* 0.45, mean rank 805/1816, 16p11.2 duplications; Additional file 3: Fig. S6). These results demonstrate that within the same EHR system, expression prediction based on common SNPs independently shows enrichment for CNV carrier-associated traits.

## Discussion

In this study, we sought to identify individual genes in the 16p11.2 and 22q11.2 regions driving brain-related disorders, as well as the impact of both the entire CNV and specific CNV genes on the medical phenome. In a novel in silico approach to CNV fine-mapping, we tested whether genetically driven predicted expression variation of the individual genes in each CNV was associated with ascertained brain-related disorders ascertained in GWAS data. We identified individual genes at 16p11.2 whose expression was associated with schizophrenia (*INO80E*), IQ (*SPN*), and BMI (*SPN*, *INO80E*) in the expected direction based on known 16p11.2 biology. We then used EHR data to detect (known and novel) traits over-represented in 16p11.2 and 22q11.2 carriers for comparison with individual gene results. Third, we used the same EHR system biobank containing over 1500 medical traits to explore the consequences of expression variation of 16p11.2 and 22q11.2 CNV genes in non-carriers, and we identified enrichment of brain-related traits as well as individual genes potentially driving carrier-associated traits. The results from the GWAS-derived and PheWAS analyses can be considered as independent ways to probe the function of CNV genes using expression imputation.

*INO80E*, the gene we identified as a driver of schizophrenia and BMI, is a chromatin remodeling gene and has rarely been considered in the context of brain-related traits [97]. Mice heterozygous for this gene have shown abnormal locomotor activation [98]. Locomotor activity in mice is a frequently used proxy for brain-related disorders including schizophrenia [99]. Our results are consistent with a previous observation that eQTLs from dorsolateral prefrontal cortex for *INO80E* co-localize with schizophrenia GWAS SNPs [100]. In addition, an analogous imputed expression-based transcriptome-wide association study observed association between *INO80E* and schizophrenia using summary statistics [101]. A third transcriptomic association study using prenatal and adult brain tissues also pointed to *INO80E* as a risk gene for schizophrenia [102]. By focusing on a specific schizophrenia-associated region, using individual-level data, and performing a conditional analysis, we have obtained additional precision and were able to fine-map the signal at 16p11.2 down to a single gene. Our study differs from Gusev et al. and Walker et al. in the expression prediction models used: we used 48 tissue models from the Genotype-Tissue Expression consortium, Gusev et al. used brain, blood, and adipose tissues from other consortia, and Walker et al. used prenatal and adult brain tissues only. The overlap in association results shows that our approach is robust to variation in predictive models. Furthermore, we find that the utilization of non-brain tissues in our analysis did not hinder our ability to detect this association. Mice with a heterozygous mutation in *Ino80e* showed increased body weight, consistent with our BMI association result for the same gene [98].

*SPN*, a gene highly associated with both IQ and BMI, is active in immune cells and is not known to play a role in brain-related disorders [103, 104]. Recently, a large genome-wide analysis of rare CNVs fine-mapped *SPN* duplications as a driver of several phenotypic categories including *behavioral abnormality* [105]. We note that the association *p* values for *SPN* are much lower than for any other genes showing association signal. This may be because our approach detected relatively few eQTLs for *SPN* (12 SNPs in two tissues), many of which overlapped with highly associated GWAS SNPs for both IQ and BMI, rather than contributing to noise.

Our results give evidence that pleiotropy is involved in the pathogenicity of 16p11.2, as opposed to a strictly “one gene, one trait” model. Specifically, *INO80E* was associated with both schizophrenia and BMI, and *SPN* was associated with both BMI and IQ. Genetic correlations of at least − 0.05 and as much as − 0.5 have been estimated for the BMI/IQ and SCZ/BMI pairs, suggesting that pleiotropy may play a general role in these disorders [106–109]. Consistent with the genetic correlations, most (8/12) eQTL SNPs in our prediction models for *SPN* drove the associations with both IQ and BMI.

While most associations we detected were in the expected direction given previous knowledge, *MVP* and *KCTD13* were associated with BMI in the opposite (positive) direction, and *YPEL3* with schizophrenia in the negative direction. We resolved the schizophrenia result by conditional analysis, where we found that *YPEL3* was associated with schizophrenia simply due to correlation with *INO80E*. For BMI, we were able to use UK Biobank data to determine that *MVP* was not an independent association with BMI, while *KCTD13* remained. For an example like *KCTD13*, we offer three explanations: these results may be false-positives due to correlation-based “hitchhiking,” they may demonstrate a limitation of our approach, or they may have a true BMI-increasing effect. First, we cannot rule out that it “hitchhikes” to statistical significance with other negatively associated genes due to correlation but does not contribute to BMI itself. Second, this result might represent a limitation of our eQTL-based method. *KCTD13* is a highly brain-expressed gene, but had no high-quality brain prediction models [50]. The direction of the eQTLs regulating *KCTD13* expression in the brain may be brain-specific, and brain may be the only relevant tissue for the effect of *KCTD13* on BMI. That is, *KCTD13* may have a strong negative correlation with BMI, but falsely appears positive due to the specific eQTLs used for expression prediction. Such tissue-specific eQTL directions of effect have been observed for at least 2000 genes [110]. Improved brain-specific prediction models will resolve this limitation. Third, *KCTD13* could have a true BMI-increasing effect. If so, the 16p11.2 region contains both BMI-increasing and BMI-decreasing genes, and the effect of the BMI-decreasing genes is stronger. Such a model is a potential explanation for the observation that duplications at 16p11.2 in mice, unlike humans, are associated with obesity [43]. One set of genes may be the more influential determinant of the obesity trait in each organism.

Our PheWAS of traits over-represented in 16p11.2 and 22q11.2 carriers served as a validation of our biobank EHR approach via detection of previously identified CNV-associated traits. Brain-related traits, such as *delayed milestones*, *mental retardation*, and *pervasive developmental disorders*, were among the top over-represented traits in both 16p11.2 and 22q11.2 CNV carriers. 22q11.2 deletion carriers were strongly associated with *cardiac congenital anomalies* and *cleft palate*, two of the hallmark features of the CNV. Even though the total number of CNV carriers within the biobank was relatively small, the strong known clinical associations were observed. At the same time, we identified novel traits that may be confirmed in larger samples of CNV carriers such as *sleep apnea* in 16p11.2 deletions and *hyperpotassemia* in 22q11.2 deletions.

Our PheWAS between the predicted expressions of 16p11.2 and 22q11.2 genes and 1500 medical phenotypic codes resulted in 17 phenome-wide significant gene-trait pairs. Some of these genes have been shown to drive similar traits in prior literature. The gene *AIFM3* at 22q11.2 was associated with *renal failure. AIFM3* is a gene in a proposed critical region for 22q11.2-associated kidney defects and led to kidney defects in zebrafish [111]. *SNAP29*, another gene associated with kidney defects in the same study, had *renal failure*, *NOS* in its top 1% phenome associations. *LZTR1* was significantly associated with *malignant neoplasm*, *other.* This gene is a cause of schwannomatosis, a disease involving neoplasms (albeit normally benign) [89]. Model organisms with defects in *PI4KA*, associated with *disorders of iris and ciliary body* in our study, showed eye-related phenotypes [90, 91]. Because few genes had any associations which were phenome-wide significant, we elected to analyze the top 1% of associations of each gene. We noticed that our gene-by-gene PheWAS recapitulated known Mendelian effects of approximately half of Mendelian genes at the 16p11.2 and 22q11.2 CNVs, including the effect of *TBX1* on the circulatory system, of *TANGO2* on glucose and epilepsy, and of *TBX6* on the musculoskeletal system at this threshold [28–30, 92–94]. There are three common SNPs at *TBX6* contributing to scoliosis (primarily in individuals who have additional disruptive mutations at the gene), and one was identified as an eQTL in our approach; perhaps an even stronger signal could have been observed if all three were included [112]. Notably, we found that clinical traits in the *mental disorders* category were over-represented in the top 1% of associations among all genes tested, and *mental disorders* was the only category significantly enriched. Some mental disorders, such as *psychosis*, were top PheWAS hits for multiple genes, but we were underpowered for rigorous independence testing. Moreover, three novel brain-related gene-trait pairs reached phenome-wide significance: *NPIPB11* and *SLX1B* near the CNV breakpoint at 16p11.2 with *psychosis*, as well as *SCARF2* at 22q11.2 with *mood disorders.* The expression of *SLX1B* is modified in 16p11.2 carriers; *NPIPB11* expression differences have not been detected in transcriptomic studies of 16p11.2 [43, 45]. *SCARF2* has recently been proposed as a driver of schizophrenia within a fine-mapping study within CNV carriers [105]. Integrating genetic information with the diagnosis of *mood disorders* in the clinical data allowed us to find a new candidate, *SCARF2*, at 22q11.2 that we were unable or underpowered to detect in the ascertained bipolar data alone.

We find that our results support the underlying hypothesis in which small changes in CNV gene expression affect risk for CNV-associated traits. In the three best-powered traits we had available—schizophrenia, BMI, and IQ—we were clearly able to prioritize individual gene(s) at 16p11.2. Similarly, we were able to detect PheWAS traits driven by small expression differences in CNV genes that were overlapping with traits in CNV carriers in the same biobank. Strikingly, we found that our gene-based PheWAS overlapped well with the carrier screen PheWAS for 22q11.2 when we found the most “CNV-like” extreme expression non-carriers. This observation validates our underlying model in which non-carriers with genetically predicted expression differences are more likely to show carrier-like traits.

### Limitations

The 16p11.2 and 22q11.2 CNVs are significant risk factors for ASD and schizophrenia, respectively, and yet no individual genes in either CNV were associated with case-control status for the associated trait in the best-powered datasets available to us. Assuming the true causal gene(s) for these disorders do exist within the CNV, limitations in our approach may preclude us from discovering them. As our predicted expressions are based on GWAS data, we end up underpowered to detect gene-based association signal where we are underpowered to detect SNP-based association signal. This is particularly true for ASD, in which the sample size is over 4 times less than that of schizophrenia. At the same time, predictive models for gene expression are imperfect; while they capture some of the *cis*-heritability of gene expression, they may not capture the entire variability of the expression of a gene (the largest single-tissue prediction *R*^2^ for our genes is 0.45, and the average *R*^2^ is 0.07). For example, the expression predictions of these genes are calculated solely using *cis*-eQTLs within 1 MB of the gene [57]. It may be necessary to consider the effect of *trans*-eQTLs to explore the genetic effect of expression variation accurately. Similarly, we have not considered *trans-*effects due to chromosome contacts, such as those that exist between the 16p11.2 region described here and another smaller CNV region elsewhere at 16p11.2 [113, 114]. Moreover, there are genes in both regions for which no high-quality models exist. If the causal gene is among the genes that cannot be well-predicted, we cannot detect this gene by our approach. One category of genes that are not represented in our study are microRNAs. 22q11.2 carriers have a unique microRNA signature, and the contribution of microRNA to 22q11.2-CNV-associated schizophrenia has been previously hypothesized [115, 116]. If the microRNAs are important regulatory elements for 22q11.2-associated traits, our approach is insufficient to detect them.

Rather than focusing on any specific tissue(s), we chose to perform a cross-tissue analysis, an approach that improves power to detect gene-trait associations and detected 16p11.2 genes associated with schizophrenia, IQ, and BMI [58]. While we might expect that brain-specific models would be best at detecting relevant genes for brain-related traits, we are limited by the amount of data available—brain tissue transcriptomes are available for fewer than half of the GTEx individuals [51]. An underlying assumption behind the use of all tissues (rather than just brain tissues) for these mental disorders is that eQTLs for our genes of interest are shared across tissues and that the same eQTLs affect the expression of a gene in the brain as in other tissues. In general, eQTLs tend to be either highly shared between tissues or highly tissue-specific, largely as a function of the gene being expressed exclusively or nearly exclusively in a single tissue [117]. The GTEx correlation of eQTL effect sizes between brain and non-brain tissues is 0.499 (Spearman) [51]. We may miss genes of interest that have brain-specific expression but not enough power to detect eQTLs. Furthermore, as these eQTLs come from adult tissues, we would miss genes where effects on brain-related traits are specific to early developmental timepoints.

A further limitation is that the variation in expression that can be modeled using eQTLs may be considerably smaller for some genes than the effect of deletions and duplications. For example, there may be a gene at 22q11.2 for which decreases in expression contribute to schizophrenia, but only when expression levels are reduced beyond a threshold, e.g., to nearly 50% of the expression levels of non-carriers. We saw an improvement in the overlap between the gene-by-gene and carrier/non-carrier PheWAS traits when we restricted our analyses to the individuals with the most extreme CNV gene expression across the region, supporting this threshold hypothesis which could be pursued in further study.

Alternatively, the overlap with carrier phenotypes observed when considering predictions across the CNV region could support a multi-gene hypothesis. So far, we have considered the effect of each CNV gene independently, when the genes may not be acting independently. A *Drosophila* model for 16p11.2 genes has shown evidence of epistasis between genes within a CNV as a modifier of phenotype [39]. If there are 16p11.2 traits in humans also driven by epistasis, our single-gene screen would not have detected the appropriate genes for those traits. Similarly, traits driven by multiple genes would be detectable in our carrier screen but not in our gene-by-gene PheWAS. Given the strong possibility that there are multiple genetic drivers for each trait, efficient ways to consider multiple genes are necessary [118, 119].

Because the CNV carrier individuals in our biobank are young (median age < 18), we do not yet know what traits might commonly occur once individuals reach older age. There were traits in our analysis that were over-represented in older CNV carriers, but difficult to interpret as they did not meet our frequency threshold, including the following: *dementia with cerebral degenerations* in 22q11.2 deletion carriers, *anterior horn cell disease* in 16p11.2 deletion carriers, and *cerebral degenerations*, *unspecified* in 16p11.2 duplication carriers*.* These findings show a need for longitudinal studies of carrier cohorts and studies of carriers in older age. Such additional data may point to additional clinical features of 16p11.2 and 22q11.2 CNV carriers.

## Conclusion

In developing our approach, we hypothesized that naturally occurring variation in gene expression of CNV genes in non-carriers would convey risk for traits seen in CNV carriers. We found that this was true for at least three 16p11.2-associated traits: BMI, schizophrenia, and IQ. Promisingly, the direction of association was generally consistent with whether the trait was found in duplication or deletion carriers. Our approach is computationally efficient, extendable to other CNV-trait pairs, and overcomes one limitation of animal models by testing the effect of CNV genes specifically in humans.

In this study, we synthesized information from both large GWAS studies and EHR-linked biobanks, benefiting from the strengths of both approaches. Psychiatric brain-related disorders such as autism, schizophrenia, and bipolar disorder have a population frequency below 5%, so large datasets specifically ascertained for brain-related disorders are better at providing sufficient statistical power for association analysis, especially when the effect of each gene is small. On the other hand, the presence of many diagnostic codes in a biobank helps identify brain-related traits that may be relevant to CNVs but not the primary reported symptoms, such as speech and language disorder. We were also able to carry out two distinct and complementary analyses using the same dataset. The presence of CNV carrier status in the EHR-linked biobank allowed us to probe the phenotypic consequences of the entire deletion or duplication. Then, we were able to test each CNV gene for association with the same diagnostic descriptions.

Our novel approach provided insights into how individual genes in the 16p11.2 and 22q11.2 CNVs may drive health and behavior in a human population. Expression imputation methods allowed us to study the predicted effects of individual CNV genes in large human populations. The incorporation of medical records into biobanks provided a way to determine clinical symptoms and diagnoses to which expression differences in the genes may contribute. We expect our ability to detect genes with this type of approach to increase in the coming years, as more individuals in biobanks are genotyped, the number of individuals contributing to large cohorts grows, and the methods to more finely and accurately predict gene expression improve. Additional experiments on our newly prioritized genes are necessary to determine their specific functional impact on brain-related disorders and to evaluate their value as putative therapeutic targets.

## Supplementary Information

**Additional file 1: Table S1.** List of genotyped discovery and replication cohorts used in the study. List of datasets used for discovery and replication of association results with sample sizes. The specific cohorts from the Psychiatric Genomics Consortium that were used for this analysis are listed. All variables from the UK Biobank that were used for replication are shown.

**Additional file 2: Table S2**. List of genes at or near 16p11.2 and 22q11.2. List of coding and non-coding genes in the CNV region, as well as flanking genes 200 kb on either side. Genes for which PrediXcan models based on GTEx v7 were available and the range of model qualities (R^2^) are noted, along with the number of tissues in which prediction models were available. Genes are annotated with their type (e.g., protein-coding, pseudogene, etc.), whether they are in the CNV or flanking, and any other names by which they may be referred in the literature.

**Additional file 3: Fig. S1-S6**. Supplementary figures.

**Additional file 4: Table S3**. Mendelian phenotypes annotated to 16p11.2 and 22q11.2 genes in PheWAS results. We compare Mendelian phenotypes annotated to 16p11.2 and 22q11.2 genes (as catalogued in OMIM) with our imputed gene expression PheWAS results. For each of the Mendelian traits, we list one or more related traits that were tested in PheWAS along with the p-value, selecting the trait(s) with the best p-value to represent. Traits that are in the top 1% of associations for individual genes are marked. This table is a proof-of-concept that our PheWAS approach can pick up known gene-phenotype associations but has not been quantified for enrichment due to the subjective nature of identifying related traits.

**Additional file 5: Table S4**: Identifying 16p11.2 and 22q11.2 cases from electronic health records (EHR**).** Keyword searches across all documents within the Vanderbilt EHR were performed to identify individuals carrying 16p11.2 or 22q11.2 CNVs. Individuals with documents containing matching keywords were reviewed manually to confirm the presence of 16p11.2 or 22q11.2 CNV. Individuals were excluded from case groups if their records included a mention of additional CNVs. Individuals within the 16p11.2 case groups were also excluded if the size of the reported CNV was 200-250 kb. Individuals within the 22q11.2 case group were excluded if the size of the CNV was smaller than 500 kb or if there was a mention of “distal” when referring to the deletion or duplication. Confirmed case numbers are listed, with the non-genotyped counts in parentheses. Non-genotyped individuals were used for downstream phenome-wide analyses.

**Additional file 6: Table S5**. Results of MultiXcan and S-MultiXcan associations between CNV genes and autism, schizophrenia, bipolar disorder, BMI, and IQ. For autism, bipolar disorder, and schizophrenia, z-scores and p-values come from a METAL meta-analysis across PGC cohorts. For BMI and IQ, mean z-scores and p-values come directly from S-MultiXcan output. Genes in each CNV are sorted by chromosomal position.

**Additional file 7: Table S6**. Comparison of association results to independent data. For each gene-trait pair, we list the original p-value, the GWAS trait(s) that we classified as most similar to a PheWAS trait, its best p-value in an independent dataset, the number of GWAS datasets that were used for this trait, and the rank of this gene within that dataset. For UK Biobank summary statistics, we have genome-wide data; for datasets with individual-level data, only 16p11.2 and 22q11.2 genes were calculated. See Table S2 for more information on datasets used.

**Additional file 8: Table S7**. Conditional analysis for independence of associations. Conditional analysis was performed on the PGC schizophrenia data, the UK Biobank BMI data, as well as two BioVU clinical trait associations (16p11.2 genes and *psychosis*, 22q11.2 genes and *morbid obesity*). For each trait, we performed MultiXcan first adjusting for a specific gene, then by leaving a gene in and adjusting all the other genes associated with that trait out. The *P*_*cond*_ reported in the text is the p-value of this gene-trait pair when adjusting for all other genes considered for conditioning for this trait, unless otherwise stated.

**Additional file 9: Table S8**. Traits over-represented in CNV carriers. The four categories of CNV carrier – 16p11.2 duplication, 16p11.2 deletion, 22q11.2 duplication, 22q11.2 deletion – were tested separately. The results for all clinical traits tested are provided. The number of cases and controls for each trait is given, as well as whether the p-value meets either Bonferroni or FDR correction. Traits in bold were represented in over 5% of carriers.

**Additional file 10: Table S9**. Top PheWAS associations of 16p11.2 and 22q11.2 genes. The top 15 associated traits for each gene, regardless of p-value, are shown. These represent the top 1% of associations among all traits tested. Genes are listed in alphabetical order, with each trait’s sample size and phecode noted [80].

**Additional file 11: Table S10**. Enrichment of clinical categories among the top PheWAS associations. The top 15 traits (codes) for each gene analyzed (n = 1470 gene-trait pairs) were divided into 17 clinical categories (observed counts column). The values in the expected counts column are calculated as 1470 * {the proportion of traits of that category tested}. For example, 159 out of 1531 codes tested were from the “circulatory system” category, so the expected counts for “circulatory system” are calculated as 1470*159/1531. The last column contains the p-value from a binomial test comparing whether the observed proportion of clinical categories is more extreme than expected.

**Additional file 12.** Supplemental Acknowledgements. Members of the Psychiatric Genomics Consortium who contributed to this work.

## Data Availability

Individual-level genotypes for Psychiatric Genomics Consortium cohorts can be obtained by applying at www.med.unc.edu/pgc/. Individual-level UK Biobank data can be obtained by application at https://www.ukbiobank.ac.uk/enable-your-research/apply-for-access. Summary-level genetic datasets used here are available to freely download from GIANT BMI (https://portals.broadinstitute.org/collaboration/giant/index.php/GIANT_consortium) and CNCR IQ (https://ctg.cncr.nl/software/summary_statistics). PrediXcan models are available to download at predictdb.org. BioVU contains protected patient health records which are available only by application through Vanderbilt University. GTEx genotypes and phenotypes are listed on dbGAP (phs000424.v7.p2). The complete results of our PrediXcan and PheWAS analyses of these datasets are available in the supplementary tables of this article.

## Abbreviations

CNV: Copy number variant
GWAS: Genome-wide association study
PheWAS: Phenome-wide association study
PheWS: Phenome-wide significant
ASD: Autism spectrum disorder
BMI: Body mass index
IQ: Intelligence quotient
eQTL: Expression quantitative trait locus
EHR: Electronic health record
SD: Synthetic derivative
