# Supplementary material for: Integration of genetic, transcriptomic, and clinical data provides insight into 16p11.2 and 22q11.2 CNV genes
Source: Genome Med. 2021 Oct 29;13:172. doi: 10.1186/s13073-021-00972-1 (PMC8557010; doi:10.1186/s13073-021-00972-1)
Supplement: Supplementary file 3 — Additional file 3: Fig. S1-S6. Supplementary figures. [file 13073_2021_972_MOESM3_ESM.pdf]

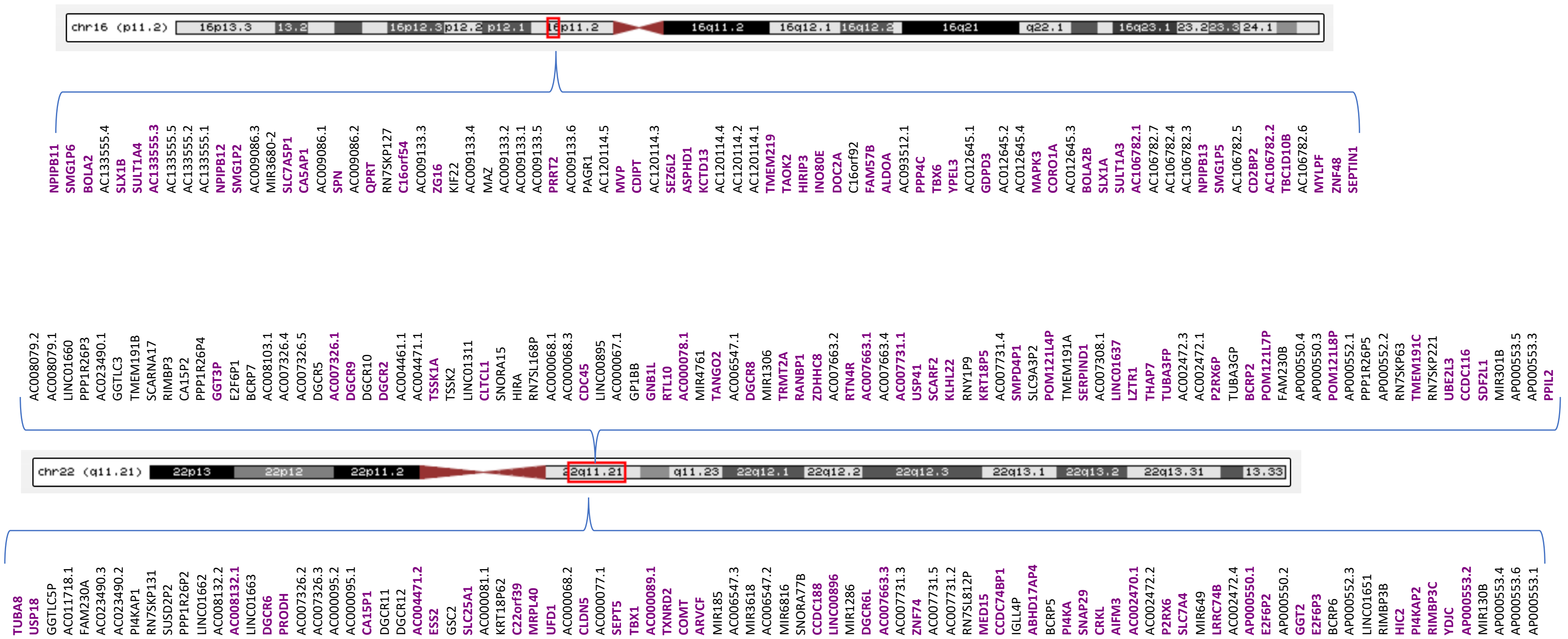

Fig S1: **Position of the 16p11.2 and 22q11.2 CNVs on chromosomes 16 and 22 and list of genes in these regions**. Bolded genes had predictive models available and were considered in our study. 22q11.2 genes are staggered on either side of the chromosome for visual clarity.

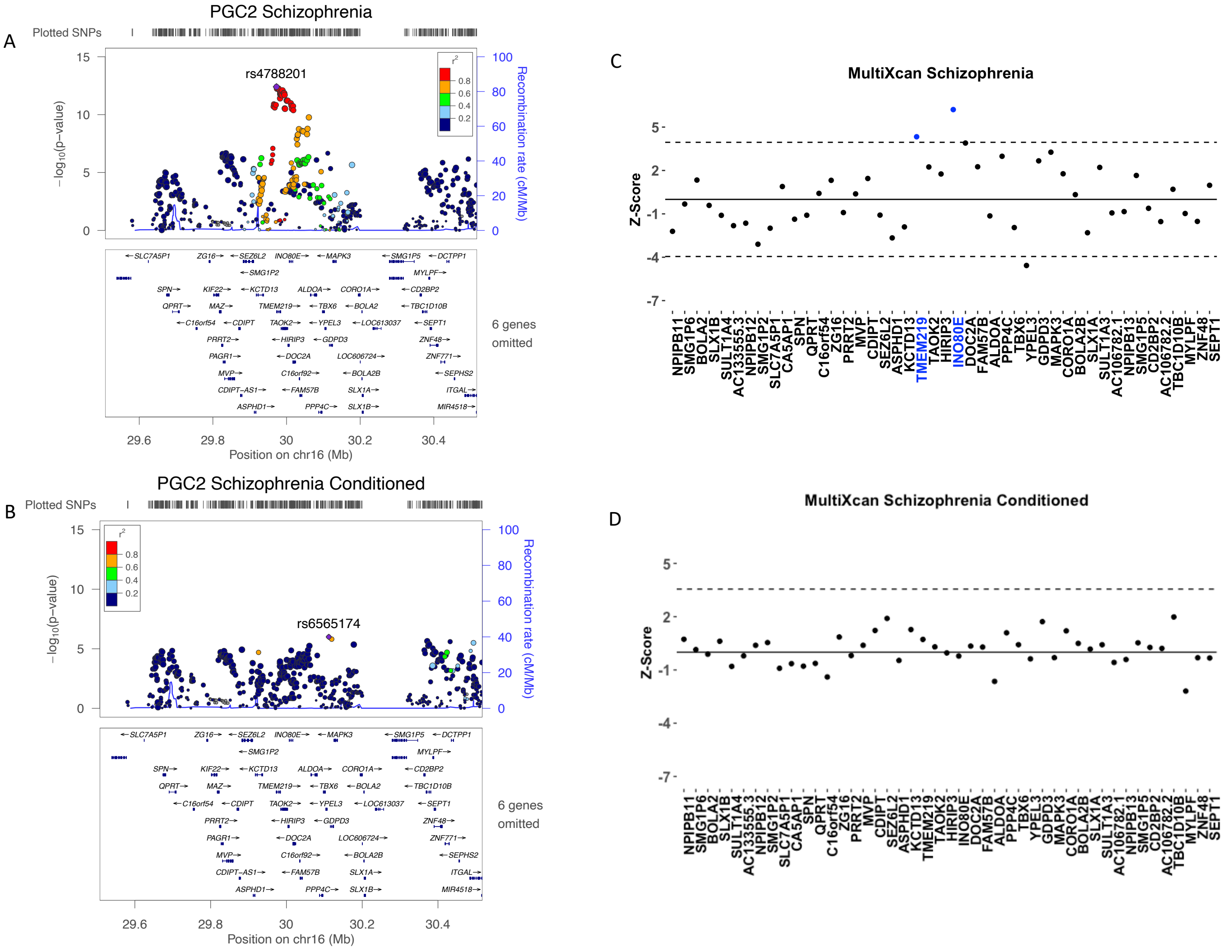

Fig S2: Conditioning MultiXcan on lead GWAS eQTL SNPs in PGC Schizophrenia and UK Biobank BMI

(A,E) LocusZoom [121] plots of GWAS summary statistics downloaded from PGC Schizophrenia and UK Biobank BMI. (B,F) GWAS conditioned on lead and eQTL SNPs; rs4788200 in schizophrenia and rs4787491, rs9936474, rs2008514, and rs8046707 in BMI. (C,G) MultiXcan gene-trait associations in schizophrenia and BMI. Significant genes are labeled in blue. Schizophrenia y-axis shows the meta-analysis z-score, BMI y-axis shows the -log10 association p-value. (D,H) MultiXcan gene-trait associations in schizophrenia and BMI after conditioning on the SNPs in (B,F).

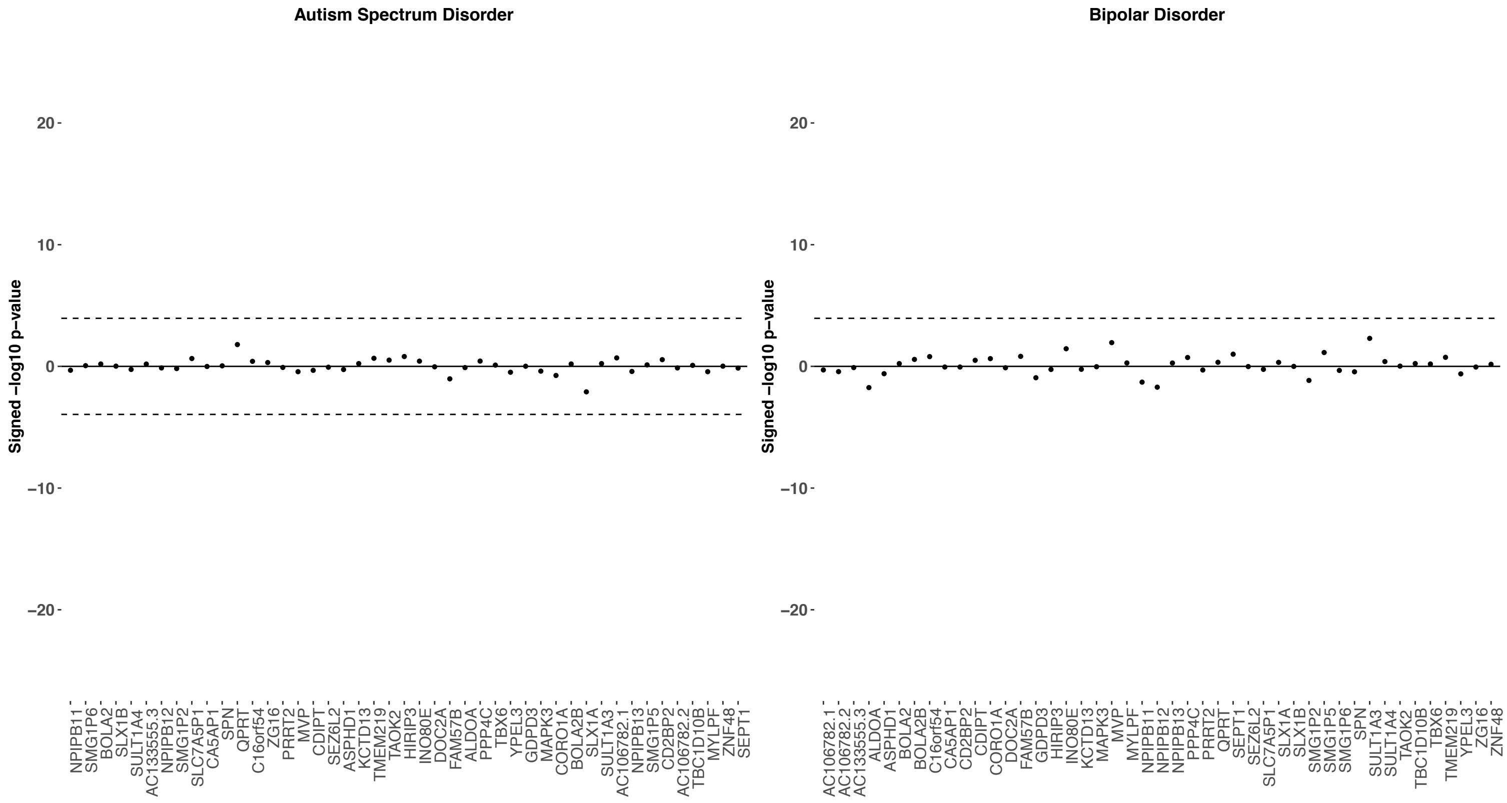

Fig S3: Association between 16p11.2 genes and the remaining two brain-related traits.

Association between predicted expression of 16p11.2 genes and ASD (left), bipolar disorder (right). Genes are listed on the horizontal axis in order of chromosomal position. The -log10 p-values on the vertical axis are given a positive or negative direction based on the average direction of the single-tissue results. The significance threshold, $P < 7.9\text{x}10^{-5}$, is a Bonferroni correction on the total number of 16p11.2 and 22q11.2 genes (127) tested across 5 traits (0.05/(5*127)).

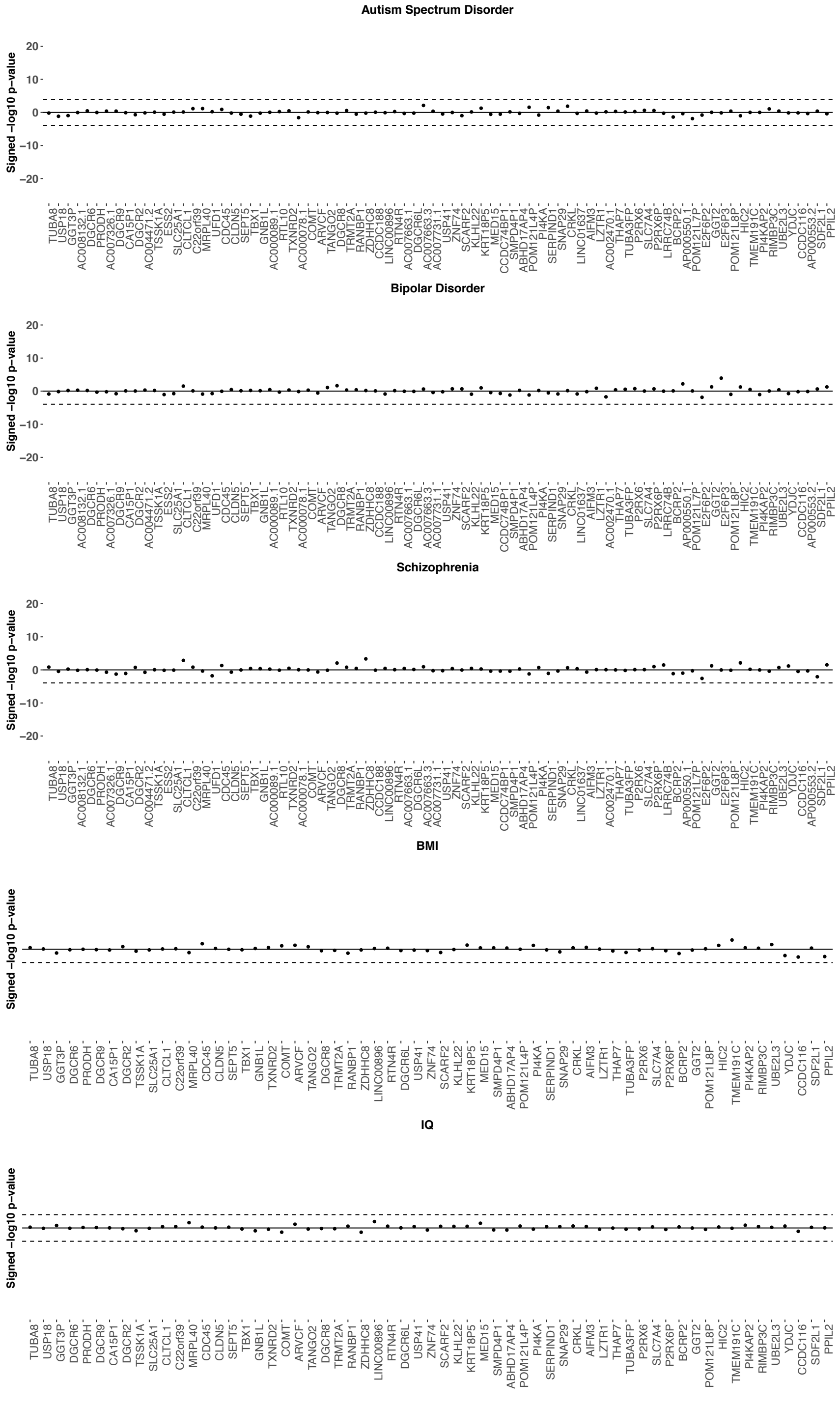

Fig S4: Association between 22q11.2 genes and five brain-related traits.

Association between predicted expression of 22q11.2 genes and, from bottom to top, ASD, bipolar disorder, schizophrenia, BMI, and IQ, using MultiXcan (ASD, bipolar disorder, schizophrenia) and S-MultiXcan (BMI, IQ). Genes are listed on the horizontal axis in order of chromosomal position. The -log10 p-values on the vertical axis are given a positive or negative direction based on the average direction of the single-tissue results. The significance threshold, $P < 7.9x10^{-5}$, is a Bonferroni correction on the total number of 16p11.2 and 22q11.2 genes (127) tested across 5 traits (0.05/(5*127)).

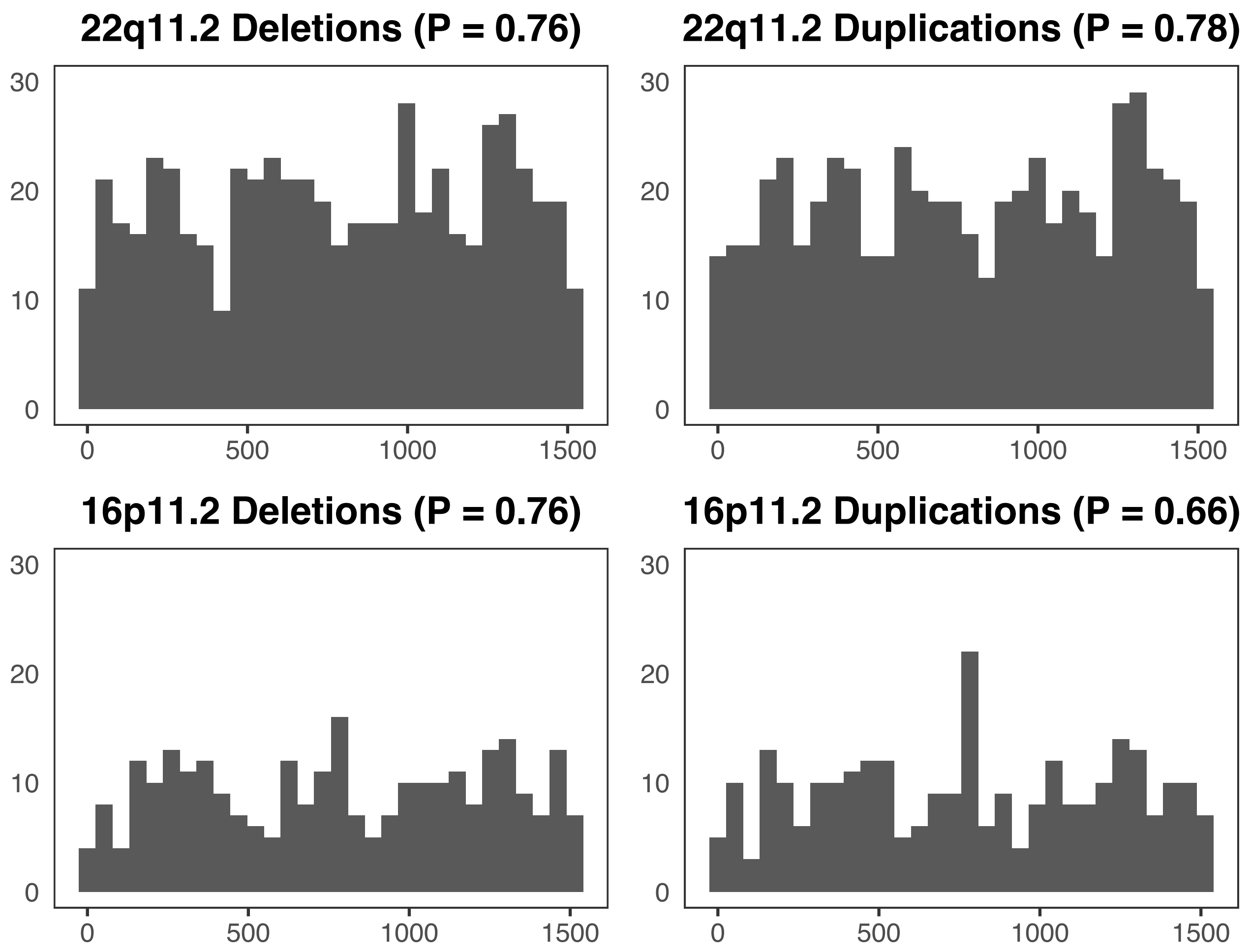

Fig S5: Test of enrichment of gene-based PheWAS results within carrier vs. non-carrier PheWAS results, using all top 1% PheWAS traits. The histograms show the distribution of ranks of the traits associated with individual 16p11.2 or 22q11.2 genes within the ranks of traits from the association analysis with carrier status. A Wilcoxon rank sum test was applied to determine whether the distribution of ranks of traits associated with individual CNV genes was different from the expected (i.e., uniform) distribution of ranks of traits associated with carrier status. The y-axis shows counts of traits from the gene-by-gene PheWAS. and the x-axis shows ranks. The gene-based PheWAS results would be considered enriched within carrier status PheWAS results if their ranks tended to skew towards one side of the distribution.

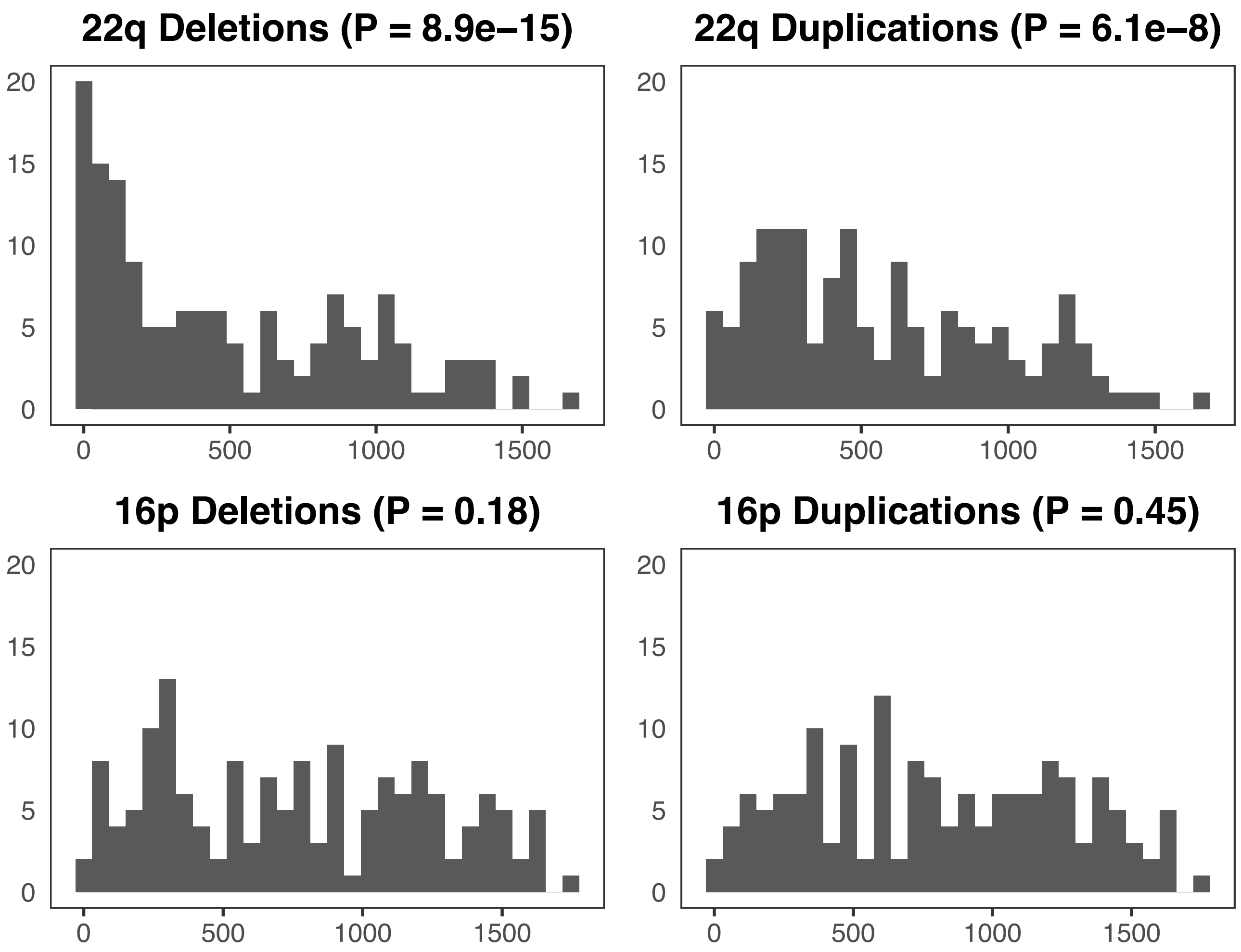

Fig S6: Test of enrichment of gene-based PheWAS results within carrier vs. non-carrier PheWAS results, using extreme-expression carriers only. The histograms show the distribution of ranks of the top 10% of traits over-represented in 16p11.2 and 22q11.2 "extreme-expression" carriers, within the ranks of traits from the association analysis with carrier status. A Wilcoxon rank-sum test was applied to determine whether the distribution of ranks of traits associated with individual CNV genes was different from the expected (i.e., uniform) distribution of ranks of traits associated with carrier status. The y-axis shows counts of traits from the gene-by-gene PheWAS. and the x-axis shows ranks. The gene-based PheWAS results would be considered enriched within carrier status PheWAS results if their ranks tended to skew towards one side of the distribution.
