# Supplementary material for: Integration of genetic, transcriptomic, and clinical data provides insight into 16p11.2 and 22q11.2 CNV genes
Source: Genome Med. 2021 Oct 29;13:172. doi: 10.1186/s13073-021-00972-1 (PMC8557010; doi:10.1186/s13073-021-00972-1)
Supplement: Supplementary file 4 — Additional file 4: Table S3. Mendelian phenotypes annotated to 16p11.2 and 22q11.2 genes in PheWAS results. We compare Mendelian phenotypes annotated to 16p11.2 and 22q11.2 genes (as catalogued in OMIM) with our imputed gene expression PheWAS results. For each of the Mendelian traits, we list one or more related traits that were tested in PheWAS along with the p-value, selecting the trait(s) with the best p-value to represent. Traits that are in the top 1% of associations for individual genes are marked. This table is a proof-of-concept that our PheWAS approach can pick up known gene-phenotype associations but has not been quantified for enrichment due to the subjective nature of identifying related traits. [file 13073_2021_972_MOESM4_ESM.docx]

| Gene | Known Clinical Traits | PheWAS Traits | PheWAS P-value | Pubmed or OMIM ID |
| --- | --- | --- | --- | --- |
| *TBX6* | Congenital scoliosis | Pathologic fracture of vertebrae^1^  Kyphoscoliosis and scoliosis | 0.0031  0.02 | OMIM: 602427 |
| *MYLPF* | Congenital contractures, scoliosis | Other congenital musculoskeletal anomalies^1^ | 0.013 | PMID: 32707087 |
| *PRRT2* | Epilepsy | Partial epilepsy | 0.14 | OMIM: 614386 |
| *ALDOA* | Hemolytic anemia Mental retardation  Muscle weakness | Acquired hemolytic anemias | 0.017 | OMIM: 611881 |
| *CORO1A* | Immunodeficiency | Varicella infection Postoperative infection Infectious mononucleosis | 0.053 0.063  0.069 | OMIM: 605000 |
| *TBX1* | Cardiovascular abnormalities | Other chronic ischemic heart disease, unspecified^1^  Endocarditis^1^  Cardiomyopathy^1^  Coronary atherosclerosis^1^ | 0.001  0.0046  0.0055  0.0076 | OMIM: 602054 |
| *TANGO2* | Hypoglycemia  Seizures | Abnormal glucose^1^  Epilepsy, recurrent seizures, convulsions^1^ | 0.0013  0.0049 | PMID: 26805781 |
| *USP18* | Cerebral calcification, hemorrhages | Cerebral edema and compression of brain^1^ | 0.00011 | PMID: 27325888 |
| *SERPIND1* | Thrombophilia | Polycythemia vera, secondary^1^ | 0.0011 | OMIM: 142360 |
| *PRODH* | Epilepsy  Mental retardation | Generalized convulsive epilepsy  Speech and language disorder | 0.067  0.14 | OMIM: 239500 |
| *CDC45* | Short stature  Ear defects  Craniosynostosis  Multiple congenital defects | Short stature  Other acquired musculoskeletal deformity  Other congenital musculoskeletal anomalies | 0.61  0.058  0.052 | PMID: 27374770 |
| *TXNRD2* | Glucocorticoid deficiency  Hyperpigmentation | Glucocorticoid deficiency  Adrenal hypofunction  Congenital anomalies of skin | 0.26  0.1  0.2 | OMIM: 606448 |
| *SNAP29* | Microcephaly  Neurologic impairment  Ichthyosis  Keratoderma | Hereditary/idiopathic peripheral neuropathy  Pruritus and related conditions  Other hypertrophic and atrophic conditions of skin | 0.61  0.078  0.24 | OMIM: 609528 |

* predictive models not available

^1^ in top 1% of PheWAS traits for this gene
