# Supplementary figures and images for: Integration of genetic, transcriptomic, and clinical data provides insight into 16p11.2 and 22q11.2 CNV genes
Source: Genome Med. 2021 Oct 29;13:172. doi: 10.1186/s13073-021-00972-1 (PMC8557010; doi:10.1186/s13073-021-00972-1)

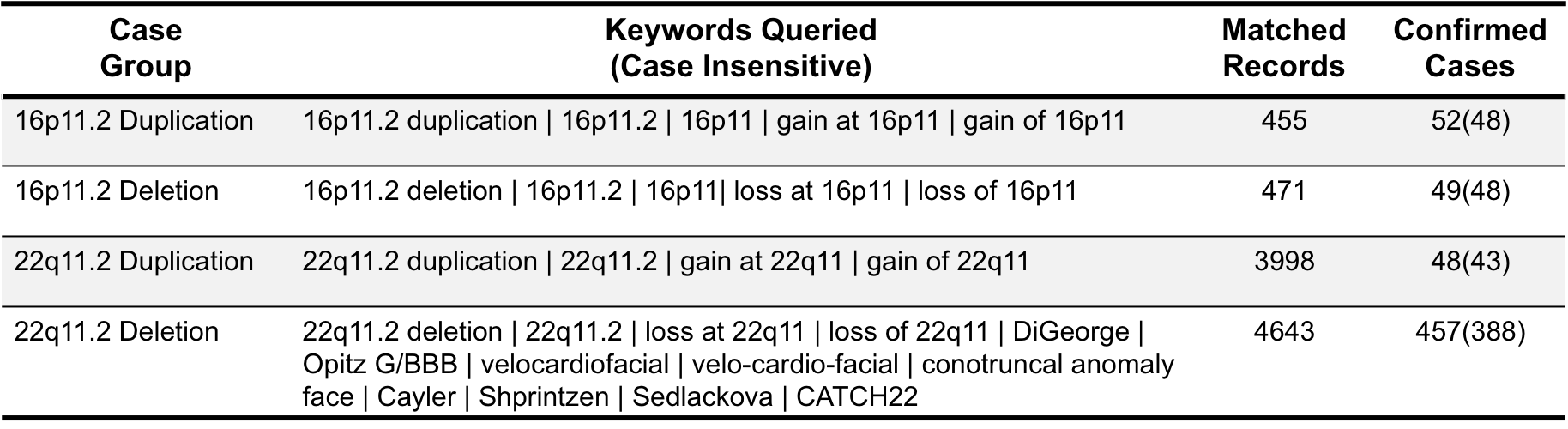

Supplement: Supplementary file 6 — Additional file 6: Table S5. Results of MultiXcan and S-MultiXcan associations between CNV genes and autism, schizophrenia, bipolar disorder, BMI, and IQ. For autism, bipolar disorder, and schizophrenia, z-scores and p-values come from a METAL meta-analysis across PGC cohorts. For BMI and IQ, mean z-scores and p-values come directly from S-MultiXcan output. Genes in each CNV are sorted by chromosomal position. [file 13073_2021_972_MOESM6_ESM.docx]
